# Exposure Modelling of Extremely Low-Frequency Magnetic Fields from Overhead Power Lines and Its Validation by Measurements

**DOI:** 10.3390/ijerph14090949

**Published:** 2017-08-23

**Authors:** Alfred Bürgi, Sanjay Sagar, Benjamin Struchen, Stefan Joss, Martin Röösli

**Affiliations:** 1 ARIAS umwelt.forschung.beratung gmbh, Gutenbergstrasse 40B, 3011 Bern, Switzerland; alfred.buergi@arias.ch; 2 Swiss Tropical and Public Health Institute, Department of Epidemiology and Public Health, Socinstrasse 57, 4051 Basel, Switzerland; sanjay.sagar@unibas.ch (S.S.); benjamin.struchen@unibas.ch (B.S.); 3 University of Basel, Petersplatz 1, 4051 Basel, Switzerland; 4 Federal Office for the Environment (FOEN), 3003 Bern, Switzerland; stefan.joss@bafu.admin.ch

**Keywords:** high-voltage power lines, magnetic fields, extremely low frequency, exposure model, measurement

## Abstract

A three-dimensional model for calculating long term exposure to extremely low-frequency magnetic fields from high-voltage overhead power lines is presented, as well as its validation by measurements. For the validation, the model was applied to two different high-voltage overhead power lines in Iffwil and Wiler (Switzerland). In order to capture the daily and seasonal variations, each measurement was taken for 48 h and the measurements were carried out six times at each site, at intervals of approximately two months, between January and December 2015. During each measurement, a lateral transect of the magnetic flux density was determined in the middle of a span from nine measurement points in the range of ±80 m. The technical data of both the lines as well as the load flow data during the measurement periods were provided by the grid operators. These data were used to calculate 48 h averages of the absolute value of the magnetic flux density and compared with modelled values. The highest 48 h average was 1.66 µT (centre of the line in Iffwil); the lowest 48 h average was 22 nT (80 m distance from the centre line in Iffwil). On average, the magnetic flux density was overestimated by 2% (standard deviation: 9%) in Iffwil and underestimated by 1% (8%) in Wiler. Sensitivity analyses showed that the uncertainty is mainly driven by errors in the coordinates and height data. In particular, for predictions near the centre of the line, an accurate digital terrain model is critical.

## 1. Introduction

Prolonged exposure to low-intensity, extremely low-frequency (ELF) magnetic fields (MF), such as those produced by high-voltage power lines, may have adverse effects on human health and has been a public health concern for several decades [1]. Since 1979, more than 30 epidemiological studies have scrutinized the association between childhood cancer and exposure to extremely low-frequency magnetic fields (ELF-MF) [2]. Pooled analyses combining the accumulating studies [3,4,5] have reported an elevated risk of childhood leukaemia associated with relatively high levels of magnetic fields exposure values from in-home measurements and calculated magnetic fields generated by overhead power lines. The strength of magnetic fields is distance dependent, and their values decrease with increasing distance from overhead power lines. Thus, several studies used distance as an exposure surrogate. For instance, Draper et al. [6] reported an increased odds ratio (OR) of 1.68 (95% confidence interval: 1.1–2.5) for childhood leukaemia among subjects living very close to overhead power lines (<50 m) compared to those residing beyond 600 m from power lines. Crespi et al. 2016 [7] reported a slight increase of cases at a distance of 50 m, with an adjusted odds ratio of 1.4 (95% confidence interval: 0.7–2.7). ORs increased very slightly for younger children (<5 years of age) and for more recent years of analysis (OR of 1.7 (95% confidence interval: 0.8–3.7) and 1.9 (95% confidence interval: 0.6–5.4, respectively). Recently, Bunch et al. 2014 [8] have re-analysed data from a previous study in relation to distance and found increased risks for children living close to overhead power lines between 1962 and 1989 but not for the later period (1990–2008). The change in the risk pattern was caused by an increase of the exposure prevalence in controls, whereas the proportion of exposed cases remained stable over time. A recent hazard assessment by the Advanced Research on Interaction Mechanisms of electroMagnetic exposures with Organisms for Risk Assessment (ARIMMORA) consortium considered the available scientific evidence published before March 2015 [9], and confirmed the previous risk assessments of IARC [10] and SCENIHR [11] that epidemiological data on the relationship of ELF-MF and childhood leukaemia is consistent with possible carcinogenicity in humans.

The epidemiological findings are relatively consistent. However, animal and toxicological studies have as yet failed to provide a biological mechanism for carcinogenicity at low exposure levels where increased childhood leukaemia risks have been observed in epidemiological studies. In addition to childhood leukaemia, there is also concern that neurodegenerative diseases [12,13] or health-related quality of life [14] may be associated with long term ELF-MF exposure from power lines.

The realization of epidemiological studies on the potential health risk from ambient ELF-MF has been hampered by the lack of a validated exposure assessment method for ELF-MF and the high cost involved with a longer time period requirement. However, the validity of exposure assessment has been an important part of studying environmental epidemiology.

The purpose of this study was to derive a model that can produce accurate long term averages of the ELF-MF from overhead lines over large areas, taking into account the diurnal and seasonal variations of the load flow for possible application in epidemiology and monitoring. Hence, a methodology and a three-dimensional (3D) computer model were developed during a pilot study in 2009 [15] and the necessary input data were identified. These data were then obtained from the electricity grid operator for a 31 km long section of the two-circuit 220 kV line Mühleberg-Bickigen/Lindenholz, for which modelling was conducted and exposure maps were calculated. In a follow-up study [16], a measurement study was carried out to validate the model at two different high-voltage overhead power lines by comparison to measured ELF-MF profiles orthogonal to the overhead lines. The model’s development and validation has also been motivated by the planned national monitoring of non-ionising radiation for Switzerland. Such monitoring has already been approved by the federal government; however, its implementation is on hold as funding is not yet secured.

## 2. Materials and Methods

### 2.1. Model

#### 2.1.1. Calculation Methods

The aim of the model was to calculate a long term time average of the magnitude of the magnetic flux density $\left|\vec{B}\right|$, e.g., an annual average using the actual line geometry and the actual operational parameters of the line.

The physical properties that enter into the calculation of magnetic fields are the current density $\vec{J}$, the magnetic vector potential $\vec{A}$, the magnetic flux density $\vec{B}$, and the magnetic field constant $\mu_{0}$. The vector potential can then be calculated [17] from

(1) $$\vec{A}\left(P_{1}\right)=\frac{\mu_{0}}{4\pi }\int \frac{\vec{J}\left(P_{2}\right)}{r_{12}}\;{\mathrm{dV}}_{2}$$

where $\vec{A}\left(P_{1}\right)$ is the vector potential in point $P_{1}$, $\vec{J}\;\left(P_{2}\right)$ is the current density in point $P_{2}$, and $r_{12}$ is the distance between points $P_{1}$ and $P_{2}$. The integration is carried out over all space. The magnetic flux density can then be calculated as the curl (a vector differential operator) of the vector potential

(2) $$\vec{B}=\nabla \times \vec{A}.$$

The magnetic field is hence calculated from an integral over the distribution of current densities in space (i.e., in the conductors) and the distance between current-carrying conductors and $P_{1}$.

For simple geometries, e.g., thin, straight wires, the integral in Equation (1) can be solved analytically [17]. In the model, the geometry of the conductors is approximated as a collection of short linear segments; the resulting field is the sum of the contributions from all the segments. For each conductor segment, the magnetic field contribution is proportional to the current I, which is the integral of the current density over the cross-section of the conductor.

The magnetic fields are three-dimensional vectors; the currents as well as the vector components have magnitude and phase. The appropriate mathematical entities to deal with such properties are complex-valued vectors.

The main input data for the modelling are geometric data and load flow data.

#### 2.1.2. Load Data

Load flow data, including magnitude, phase, and load flow direction for each current circuit, are needed to calculate the active current $I_{a}$ and reactive currents $I_{r}$. The load flow data are recorded by the operators of the electricity grid, typically as time, voltage U (in kV), active power P (in MW), and reactive power Q (in MVAR). The calculation is done as following:

(3) $$P+iQ=\sqrt{3}\;U\;I^{*}=\sqrt{3}U\;\left(I_{a}–\;i\;I_{r}\right)$$

for a symmetrical three-phase system, where the asterisk * denotes complex conjugation and i the imaginary unit.

**Figure 1:**
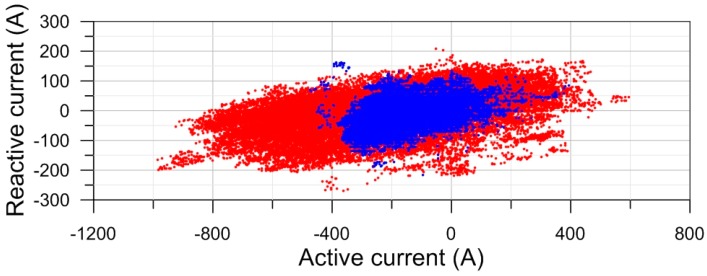
Scatterplot of active and reactive currents on a two-circuit 200 kV/132 kV line, 15 min averages for one year. Red: 220 kV, blue: 132 kV. Source: report on the pilot project [15].

**Figure 2:**
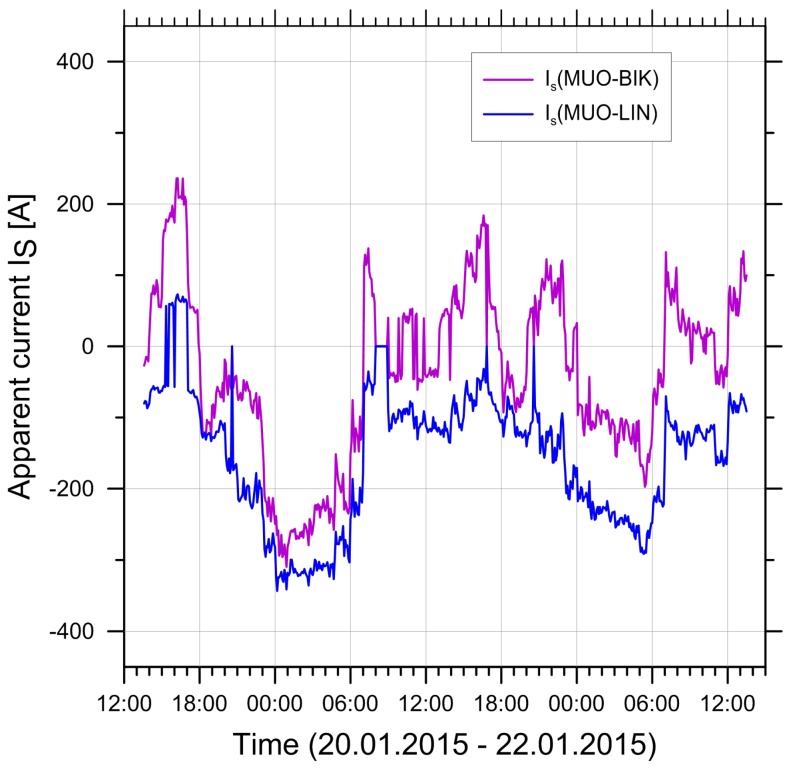
Daily variation of current data in two circuits of a 220 kV/220 kV line. Data from the first measurement period. The colours distinguish the two circuits. MUO-BIK, Mühleberg-Ost to Bickigen; MUO-LIN, Mühleberg-Ost to Lindenholz.

The currents show strong daily and seasonal variations. On lines with multiple circuits, they are in general different on different circuits, they can change direction, and also the relative directions between circuits can change from same-direction to opposite. This is illustrated by the data from the pilot study shown in Figure 1 for the range of current variations over an entire year and in Figure 2 for the variations over a 48-h period.

The resulting field at any location is the vector sum of the contributions of the different circuits. Depending on the geometry, the phase assignment, and the varying relative directions of the load flow, the fields can either amplify or partly compensate.

**Figure 3:**
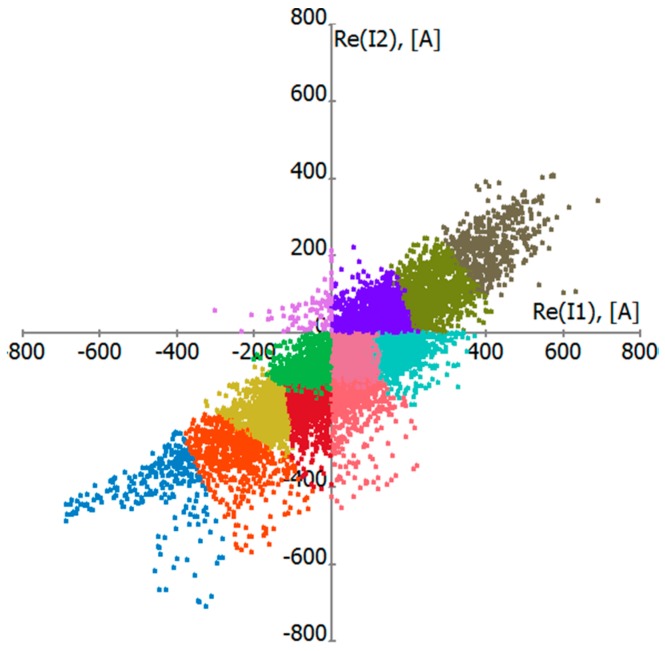
K-means-clusters for a 2 × 220 kV line with k = 12 clusters. Projection of the clusters on the plane of the active currents (i.e., real part of complex current). Points belonging to different clusters are distinguished by different colours.

Since the temporal variation of the load data is high, these computations need to be done with a high temporal resolution (15–60 min). To accelerate the calculation of time averages, the load data were grouped in clusters of similar values. This grouping can be automated by a procedure called k-means-clustering [18]. The method is iterative: each cluster is represented by its centre point. Starting with a small number of clusters with arbitrary initial values, data points are assigned to the cluster with the closest centre. When all of the points are assigned, the cluster centres are updated, and, while the number of clusters is smaller than the intended number k, the cluster with the largest variance is split in two. The steps (assignment, update, and possible split) are repeated until the assignments no longer change. The algorithm always converges to a fixed point. In our implementation, the points are always assigned to clusters with the same sign in all parameters to avoid the cancellation of positive and negative values. For n circuits, the clustering is carried out in 2n-dimensions (active and reactive current for each circuit). An example plot for hourly load data from the pilot study using a clustering with k = 12 is shown in Figure 3.

#### 2.1.3. Geometric Data

**Figure 4:**
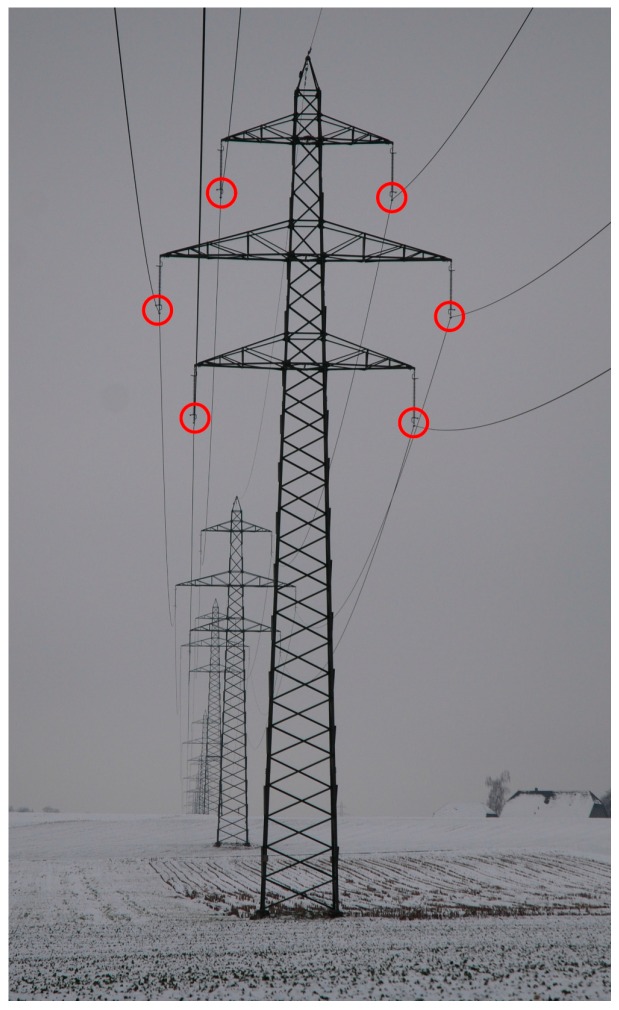
Position of the conductors on the tower, case of the 2 × 220 kV line in Iffwil. The position of the attachment point is determined by the position and geometry of the tower and the length of the isolators.

The geometric data describe the position of the towers and their geometry, i.e., the lateral and vertical position where the conductors are attached (as shown in Figure 4), the line sag between towers, the phase arrangement of the conductors, and finally one also needs the topographical data of the area surrounding the line in the form of a digital terrain model.

Of the geometric data, only the line sag and lateral displacement by wind are variable in time. Line sag depends on a number of properties (weight, elasticity, thermal expansion coefficient, and tensile stress), the temperature, the span between towers, and added load (e.g., ice, wind). The conductor temperature depends on Ohmic heating by the current, the meteorological conditions (air temperature, wind and solar irradiation), and radiative cooling. The maximum sag is found at the maximum conductor temperature (maximum current) or the maximum added load. Under most circumstances, the currents are smaller and the cooling more efficient than in the maximum sag case, which means that the temperature is not much higher than the air temperature (this would no longer be true if the line is operated near its maximum capacity or in case of so-called high-temperature conductors, these cases would have to be treated differently). For the lines under study, with spans of approx. 300 to 400 m between towers, the sag, as given by the tables of Swissgrid and Bernische Kraftwerke (BKW), varies by only about ±1 m for a temperature variation of ±30 °C (i.e., between −20 °C and +40 °C), and thus a constant approximate value is applied in the model, which makes the sag, and therefore the line geometry, independent of the time interval. Lateral displacement by wind is neglected in the model.

**Figure 5:**
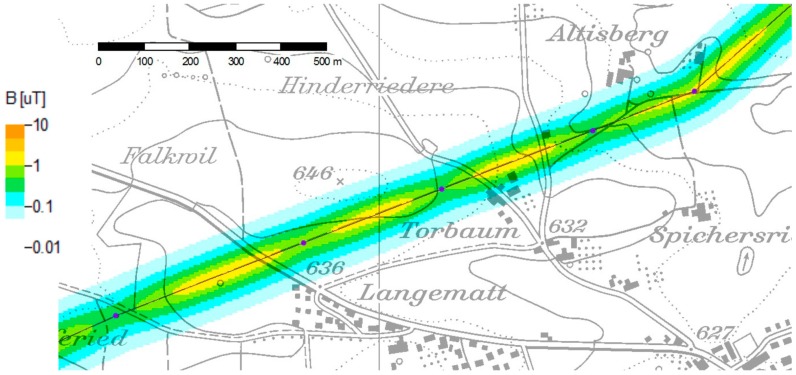
Detailed result of a color-coded magnetic field map produced in the pilot project. Background map PK25 © Swisstopo.

Figure 5 shows in an exemplary way the effects of line sag and topography for the pilot study modelling [15]. Elevated field strengths are found only in narrow corridors around the power line, and are typically highest in the middle between two towers.

#### 2.1.4. Error Estimates and Simplifications

In this section, some error estimates will be made based on a simple two-dimensional approximation, and some possible simplifications will be discussed based on these estimates.

The field of a single thin, straight, infinitely long conductor is given as

(4) $$B=\frac{\mu_{0}I}{2\pi r}.$$

Here, *I* is the current and *r* is the distance from the conductor. For a circuit of three-phase conductors, the corresponding field strength is approximately, for r≫g

(5) $$B=\frac{\mu_{0}}{2\pi }\;\frac{Ig}{r^{2}}$$

where g is a geometry factor which has the dimension of length. It represents the mean distance between the conductors, which depends on the geometrical arrangement of the conductors. Example values for various configurations can be found in the Electric Power Research Institute (EPRI) “red book” (Table 7.4.3 of [19]). 

Directly under a line, the field is most strongly influenced by the lowest conductor, and the distance is approximately r≈h, i.e., the height of the lowest conductor. Then, the field is approximately given by (4), and the uncertainties δB and δh are related by

(6) $$\frac{\delta B}{B}\approx \frac{\delta h}{h}.$$

In this case, an error of 1 m for a line at height 10 m would give a relative error of 10% in both h and B. At larger horizontal distances from the line centre, the field of a three-phase circuit scales as in Equation (5), with the distance r approximately equal to the horizontal distance. As the field scales as $1/r^{2}$, an error δr in distance produces an error of the field

(7) $$\frac{\delta B}{B}\approx 2\frac{\delta r}{r}$$

hence, a 10% position error of 5 m at distance 50 m would lead to a 20% error in the magnetic field. Large positional errors could possibly occur in epidemiological studies when home addresses are converted into coordinates, as has also been noted in [20].

**Figure 6:**
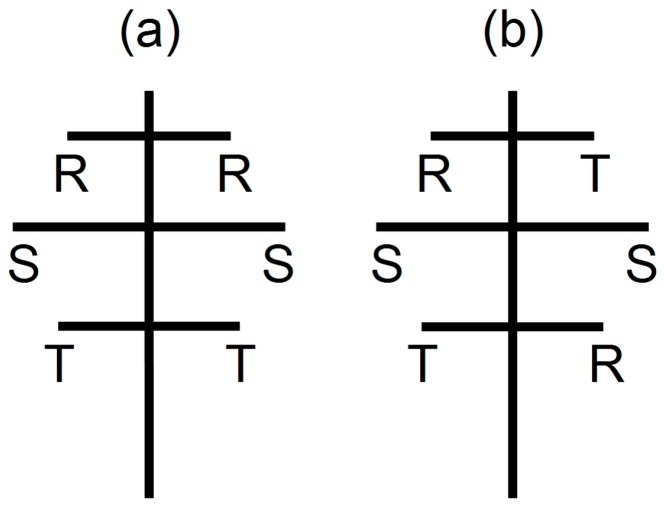
Two common phase arrangements for a standard 220 kV or 380 kV tower, for phases designated R, S, and T. (**a**) plane symmetry; (**b**) central symmetry. Four other, intermediate configurations are also possible, but not shown here.

For a double-circuit line and towers as in Iffwil or Wiler, different phase arrangements of the two circuits are possible, and the two most common configurations are shown in Figure 6.

The field for a line of two circuits at horizontal distance *d* and phase arrangement as in Figure 6 (or any permutation of R, S, T) can be approximated as

(8) $$B\approx \frac{\mu_{0}g}{2\pi }\;\left(\frac{I_{1}}{{r_{1}}^{2}}\pm \frac{I_{2}}{{r_{2}}^{2}}\right).$$

The plus sign applies to configuration (a), the minus sign to configuration (b) of Figure 6. Assuming equal currents $I_{1}=I_{2}=I$ in both circuits, setting $r_{1,2}=r\mp d/2$, and expanding the expression w.r.t. 1/r, one obtains

(9) $$B_{p}\approx \frac{\mu_{0}g}{\pi }\frac{I}{r^{2}}\;(\mathrm{configuration}\;a,\;\mathrm{plane}\;\mathrm{symmetry},\;r\gg d$$

and

(10) $$B_{c}\approx \frac{\mu_{0}gd}{\pi }\frac{I}{r^{3}}\;\left(\mathrm{configuration}\;b,\;\mathrm{central}\;\mathrm{symmetry},\;r\gg g,d\right).$$

Depending on the phase arrangement, the magnetic field falls off either as $r^{-2}$ or as $r^{-3}$. In the first case, the magnetic fields of the circuits add up and roughly double the field; in the second case, they partly compensate and the field falls off much faster with distance. The ratio of the two field expressions with different phase symmetry is

(11) $$r=B_{p}/B_{c}\approx \frac{r}{d}\;\left(r\gg d\right).$$

Hence, for a typical 220 kV line with d≈10 m, at 50 m distance from the line the two Expressions (9) and (10) would differ by a factor of 5. In the case where the currents in the two circuits are equal and opposite (i.e., opposite load flow directions), the role of the two-phase arrangements reverses, and the fields compensate for configuration (a) and add for configuration (b).

Figure 6 and Equations (8)–(11) illustrate the difficulty of modelling magnetic fields for lines with more than one circuit. Not only is it mandatory to know the phase arrangement, but also the currents and both load flow directions must be known, since a false arrangement or load flow sign could lead to large errors, as in Equation (11). The situation becomes even more complex if currents vary in time and load flow directions change, as shown, e.g., in Figure 2, especially if they also change from parallel to antiparallel. For this reason, it is very difficult to make simple approximations for magnetic field calculations for lines with two or more circuits, except in simple circumstances.

#### 2.1.5. Computations for Validation

The model was applied to the two different measurement sites to calculate 48 h ELF-MF for each measurement period. For these calculations, the following data were obtained and used for the modelling:

- List of the coordinates of tower positions x and y (and later also z)
- Mast-schemas for all masts, one drawing per mast
- Drawings of isolators
- Phase-allocation schema for all circuits
- Graphics/tables on line sag, for all spans
- Load flow data (time, voltage, active power, and reactive power) as 15 min averages for the measurement periods and 1 h averages for the whole year 2015 from Swissgrid and in part also from BKW.
- Two different digital terrain models: (1) the DHM25 with 25 m resolution, and (2) the more precise model DHM5 with 5 m resolution.

For the calculation of the active current $I_{a}$ and reactive current $I_{r}$, we used a clustering with k = 16, which was demonstrated in the pilot study [15] to be sufficiently accurate: for the relevant heights of 10 to 20 m below the lowest conductor and all lateral distances, the relative errors were in the range of ±2%. Larger errors (up to 5%) only occurred in the near region between the conductors. The effort could even be reduced further by using root-mean-square (RMS) averages, as detailed in [16]. For comparison, these RMS averages were also calculated for the validation study.

The following approximations were therefore used for the model calculations. First, the currents are assumed to be symmetric, all phases of a circuit have the same amplitude, and the phases are shifted by exactly 120°. No account was taken of currents induced in the earth or the shield wires, nor of unbalanced currents induced in the circuits. Second, the line sag was assumed to be the line sag at 10 °C, close to the average air temperature (9 °C in the Swiss plateau).

Unless the terrain is absolutely flat, which at least in Switzerland is seldom the case, a numeric terrain model must be used to derive the height of tower bases and the height of receptor points. Two different such models were used. First, we used a relatively coarse grid of 25 m resolution (DHM25). Such grids are readily available almost everywhere, and they require small storage space and come at a low cost; however, their precision is limited. Second, we used a higher resolution (5 m) grid (DHM5). Higher resolution grids are more precise, but they come at a larger cost and may not be available everywhere.

Four different variants of the model were calculated for the comparison with the measurements, differing by the type of temporal average calculated and the precision of the terrain model:

- Model A: gives the arithmetic average $\bar{B}$ using the 25 m resolution terrain model DHM25.
- Model B: gives the RMS mean $B_{\mathrm{RMS}}$ using the 25 m resolution terrain model DHM25.
- Model C: gives the arithmetic average $\bar{B}$ using the 5 m resolution terrain model DHM5.
- Model D: gives the RMS mean $B_{\mathrm{RMS}}$ using the 5 m resolution terrain model DHM5.

The arithmetic mean $\bar{B}$ of the absolute value of the magnetic flux density $\left|\vec{B_{i}}\right|$ for n time periods was computed as the following:

(12) $$\bar{B}=\frac{1}{n}\;\sum_{i=1}^{n}\left|{\vec{B}}_{i}\right|$$

and the root-mean square $B_{\mathrm{RMS}}$ as

(13) $$B_{\mathrm{RMS}}={\left(\frac{1}{n}\;\sum_{i=1}^{n}{\left|{\vec{B}}_{i}\right|}^{2}\right)}^{1/2}.$$

There exists a simple relation between the two means, which follows directly from the definition of the variance:

(14) $$B_{\mathrm{RMS}}^{2}={\bar{B}}^{2}+\frac{n-1}{n}\;\sigma_{B}^{2}$$

where $\sigma_{B}^{2}$ is the variance of the $\left|\vec{B_{i}}\right|$.

When calculating averages as $\bar{B}$ or $B_{\mathrm{RMS}}$, one must keep in mind that the original measurements themselves always represent RMS values, as the arithmetic mean over a sinusoidally varying quantity is zero and only an RMS value is meaningful. The difference in the averaging procedure applies only to the averaging over the set of larger time periods (e.g., 15 min or 1 h values).

The calculations were carried out with an ad hoc modified version of the NISMap-software (NISMap-ELF) developed by ARIAS.

### 2.2. Measurements

#### 2.2.1. Selection of Sites for Validation

**Figure 7:**
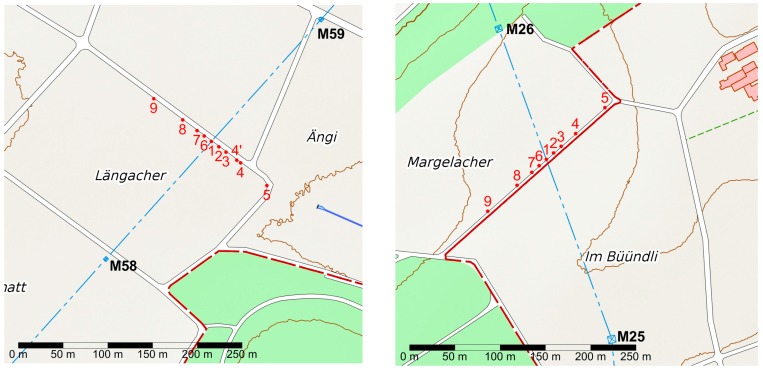
Measurement sites in Iffwil (**left**) and Wiler (**right**). The blue dash-dotted line marks the power line axis between the towers; the nearest towers are marked M58/M59 and M25/M26, respectively. The red points mark the measurement points, numbered 1 to 9. Measurement point 1 is below the line axis, points 2 to 5 are on one side, and 6 to 9 on the other side of the axis. The red lines mark boundaries between municipalities. Background map data AV, © Swisstopo.

The aim of the validation study was to validate the model by measuring at two different sites below two different overhead power lines. The first site was in Iffwil, approximately 13 km North-North East of Bern. The site is below the 2 × 220 kV line that had already been the subject of the pilot study. The line is operated by Swissgrid (during the pilot study it was still operated by BKW); it connects Mühleberg-Ost (MUO) to Bickigen (BIK) and Lindenholz (LIN). The line has single conductors (per phase), and the height of the lowest conductors above ground is approximately 12 m (at 10° conductor temperature). The second site was in Wiler bei Seedorf, approximately 15 km Northwest of Bern, below a 220 kV/132 kV line operated by Swissgrid (the 220 kV circuit) and BKW (the 132 kV circuit), connecting Mühleberg-Ost (MUO) to Pieterlen (PIE, 220 kV) and Kappelen (KAP, 132 kV). The geometry of the towers is as for a 380 kV line. The towers of the line are symmetric, but the 220 kV line has a bundle of four conductors and the 132 kV line a bundle of two conductors. As the tower to the north of the site is situated at the edge of a wood, it is much higher than the southern one, and the minimum ground distance of the conductors is to the south of the measurement site. Consequently, the ground distance of the lowest cables is higher in Wiler, with a nominal 20.8 m (220 kV) and 19.5 m (132 kV) at 10 °C. The differences are due to different isolator lengths and the inclination of the terrain. Both sites are in rural settings on agricultural paths where very few people pass. The measurements were made along the agricultural paths, which pass below the power line at near right angles (84.6° in Iffwil, 68.5° in Wiler). Maps of the sites and the measurement points are shown in Figure 7.

#### 2.2.2. Measurement Procedures

To capture daily variations, each measurement lasted for 48 h during each measurement period. To capture seasonal variations, measurements at each site were repeated approximately every two months during one year. This resulted in a total of 12 measurement periods, six each at each measurement site (Iffwil and Wiler), in the period between January and December 2015. The measurements were carried out during the week, typically from Tuesday to Thursday (only one period was from Wednesday to Friday, in the week after Easter). The measurement periods are listed in Table 1. The measurements typically started around noon, somewhat earlier in summer to avoid the heat, and somewhat later in fall and winter to avoid the morning fog.

Measurement devices were placed in a lateral transect at orthogonal distances *D* = 0, ±10 m, ±20 m, ±40 m, and ±80 m from the centre of the line. This profile was located approximately in the middle of a span. The magnetic field measurement devices were placed at approximately 10 cm above ground.

It was ensured that no other electricity lines or communication cables were located in the vicinity of the measurement points. However, in Iffwil, the village brook is flowing in a duct underneath the measurement path, and one of the points in Iffwil (Point 4) was very near to a manhole cover of this village brook. During the setup for the first measurement period, the ground was snow-covered and the manhole had remained unnoticed. The cover was of cast-iron and concrete. Due to this, the point was shifted by 5 m (to Point 4) after the first measurement. The magnetic field measurement devices were the following:

- Emdex II. Seven devices were placed at distances 0 to ±40 m.
- Estec DL-MW10s. One device was placed at distance 0 m together with an Emdex II, and a second one was used as a spare and for spot measurements.
- Estec EMLog2e. Two devices were placed at ±80 m, as they have higher sensitivity and resolution than the Emdex II and DL-MW10s.

The technical data of the devices are given in Table A1 of Appendix A. The parallel measurements of the EMDEX II and Estec devices agreed to within 2% in Iffwil and 0.5% in Wiler, on average, corresponding well with the indicated precision (3%) specified for both types of devices. The Emdex devices had been repeatedly calibrated in a previous measurement campaign [21] and found to be very stable. The Estec devices were new and had been calibrated by the manufacturer.

**Figure 8:**
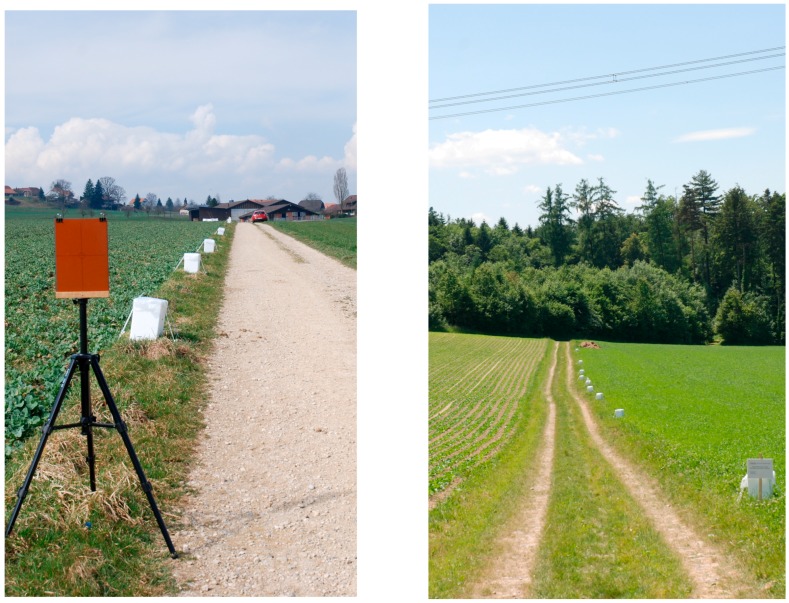
Measurement setup with the (white) measurement boxes in Iffwil (**left**) and Wiler (**right**). Only the right image shows the entire length of the measurement path with all nine boxes. Also shown in the left picture is the target table that was used with the laser distance meter to position the boxes and to measure the height to the conductors.

All magnetic field measurement devices are specified for positive temperatures only (0 °C to 60 °C for the Emdex devices and 0 °C to 40 °C for the Estec devices). In order to protect them from temperatures outside the specified range, they were placed in thermally isolating boxes (of expanded polystyrene, EPS) and thermally buffered with a phase-change material (PCM) with a phase transition at 5 °C. The PCM used was PX05 (manufacturer Rubitherm, www.rubitherm.com), which is composed of a mixture of paraffin and water embedded in an inorganic matrix. It comes in the form of a crystalline powder, is chemically inert, nonconducting, and nonmagnetic. Some 325 g of PX05 were placed in every measurement box. In order to verify that the temperature in the boxes remained indeed in the allowed range, temperature loggers (Rotronic TL-1D) were used in two of the boxes. The measurement boxes were protected against the weather with additional plastic covers and fixed to the ground with plastic straps. All materials used were nonmetallic, nonconductive, and nonmagnetic. The setup of the measurement boxes at both sites is shown in Figure 8.

## 3. Results

### 3.1. Pilot Study

The results of the feasibility and pilot study [15] were the identification of the necessary input data and the development of the methodology to calculate long term averages of the magnetic field. An estimate of the work effort required for the data acquisition resulted in at least 4.5 to 6 work hours for an equivalent line of some 30 to 40 km length with some 100 towers once the basic model is set up and the contact to the grid operator established. With some 100 such lines in the Swiss transmission system (220 kV and 380 kV lines), this would already result in a considerable effort for a complete exposure map. Compared to this, the actual computational effort on the computer is negligible.

Finally, an error estimate was performed and the precision of the results estimated to approximately 10 to 25%. The largest errors result from uncertainties in the coordinates, i.e., the x-y-coordinates, the height (both of conductors and terrain), and the line sag.

### 3.2. Modeling

**Figure 9:**
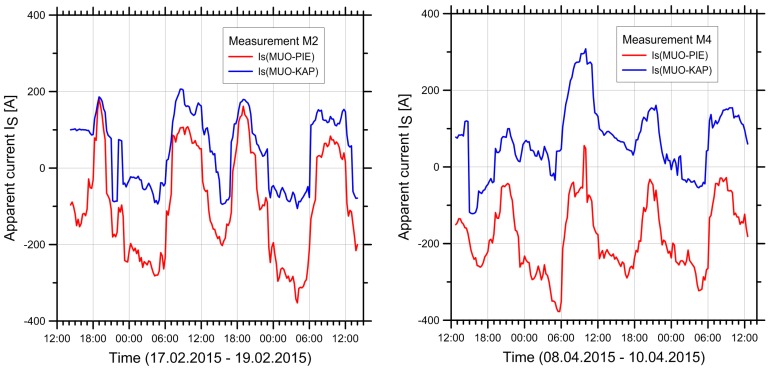
Plots of the apparent current $I_{S}$ in the two circuits MUO-PIE (Mühleberg-Ost to Pieterlen) and MUO-KAP (Mühleberg-Ost to Kappelen) during measurement periods M2 (**left**) and M4 (**right**) in Wiler.

For every measurement period, the load flow data were obtained from the grid operator and the currents were calculated. Examples of the typical behaviour of the currents on the two circuits are shown in Figure 9 for measurement periods M2 and M4 in Wiler. During both periods, a high correlation of the currents in the two circuits is visible. The figure also shows the peaks that occur at certain daytimes. Also visible from the figure is the change in the currents’ sign (i.e., the change in the direction of load flow), but also that the currents in the two circuits sometimes have the same sign (most of the time during M2), but sometimes also have opposite signs (most of the time during M4).

### 3.3. Measurements

**Figure 10:**
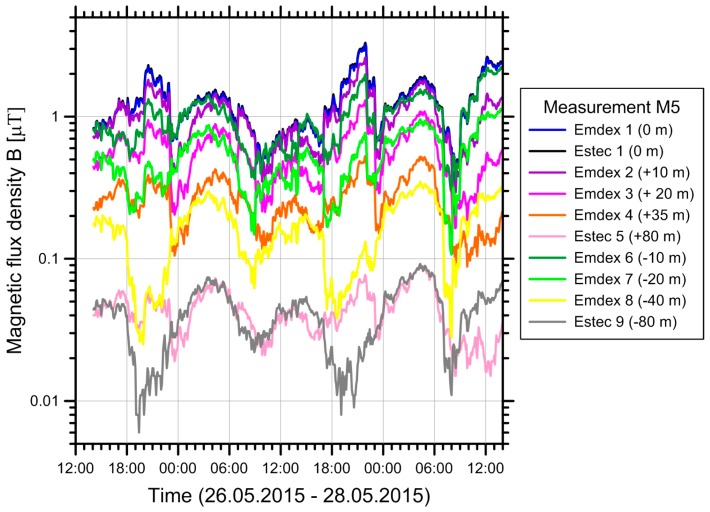
Measured magnetic fields during measurement M5 for all devices. The devices Emdex 1 and Estec 1 were measuring at the same measurement point and the curves of these devices are indistinguishable in the figure.

For each measurement period, a time series of the magnetic field at each measurement point was collected. An example, for measurement M5, is shown in Figure 10. The magnetic flux densities range from maxima of around one to a few µT near the line axis down to less than 10 nT at the outermost measurement points. The curves on the two sides of the line centre (positive vs. negative distances) show somewhat different behaviour, reflecting different loads on the two circuit systems.

**Figure 11:**
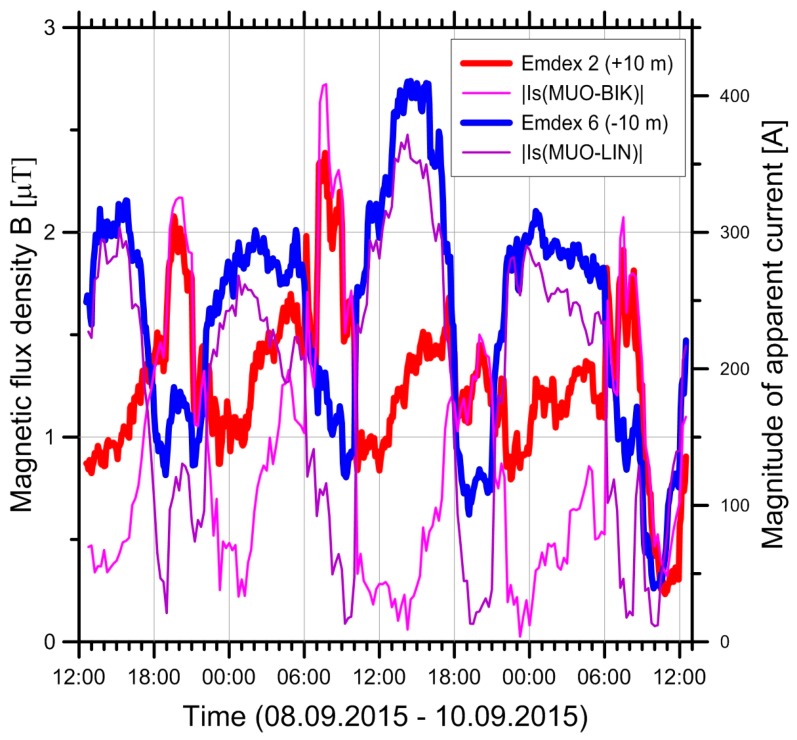
Magnitude of apparent current in the two circuits MUO-BIK and MUO-LIN and magnetic fields measured at ±10 m (Emdex 2 and Emdex 6, measurement M9 in Iffwil). Emdex 2 measures on the side of MUO-BIK, Emdex 6 on the side of MUO-LIN.

Figure 11 shows an example of a temporal pattern between the apparent currents $I_{S}$ in the two circuits and the magnetic fields measured at ±10 m from the measurement period M9. It is visible that the magnetic field follows closely the current on the nearer circuit, at least as long as one of the currents is dominant.

### 3.4. Comparison between Measurements and Modelling

**Figure 12:**
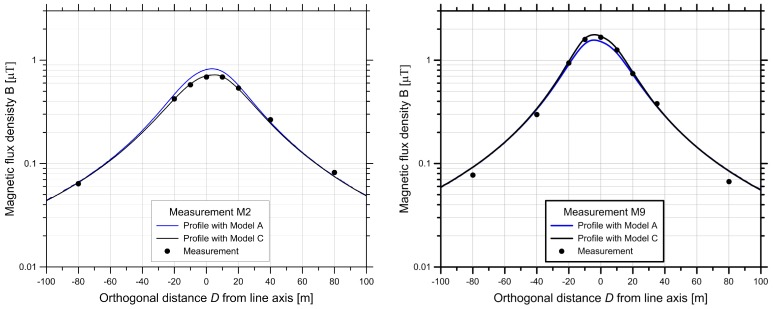
Comparison of the modelled lateral profiles for two measurements. (**Left**) M2 in Wiler; (**Right**) M9 in Iffwil. The dots are the measurements, and the lines are the calculated values for two different numerical height models: Model A uses the DHM25 (blue curves), Model C uses the more precise DHM5 (black curves).

The comparison of the measured magnetic arithmetic means $\bar{B}$ and Models A and C is shown in Figure 12 for two of the measurement periods. It can be seen that the Model C based on the more precise digital terrain model DHM5 (5 m resolution) yields better agreement for the points near the line axis compared to Model A (DHM25). For measurement points further away from the centre line (>30 m), the two models provide nearly the same results. The difference between the two models is more pronounced in Wiler, since one of the towers is standing on a ridge. This made the interpolation with the DHM25 imprecise. The height of the conductors above the path, as derived from the numerical terrain model, was different by about two meters between the two models. Measurements with a LASER distance-meter revealed that the DHM5 model provided correct values for the distances of the conductors above ground, but the DHM25 model did not. In the case of measurement M9, the largest relative deviations between model and measurement occurred at the largest distances (±80 m).

The measured and calculated time averages for all measurement periods and all measurement points are given in the tables of the supplementary material.

#### Statistical Evaluation of Model Uncertainty

As the magnitude of the measured values varies by orders of magnitude between near and far points, it is reasonable to analyse the relative deviation between model and measurement, defined as

(15) $$\Delta \;:\;=\frac{B\;\left(\mathrm{model}\right)-B\left(\mathrm{measured}\right)}{B\left(\mathrm{measured}\right)}.$$

Depending on the model, B is the arithmetic mean $\bar{B}$ (Models A and C) or the RMS mean $B_{\mathrm{RMS}}$ (Models B and D). The results are given as mean M(Δ) (offset) and standard deviation σ(Δ) for all measurement points at a measurement site and a given model variant; they are tabulated in Table 2.

On average, Model A underestimates the magnetic fields in Iffwil by 4% (standard deviation SD: 11%) and overestimates them in Wiler by 7 to 8% (SD: 8%). According to Model C, the mean deviation in Iffwil was 2% (SD: 9%) and in Wiler 1% (SD: 8%) (Table 2). Independent of the terrain model used, the results for the relative deviation Δ are very similar for $\bar{B}$ or $B_{\mathrm{RMS}}$ (Model A vs. Model B and Model C vs. Model D).

The three most pronounced discrepancies between model and measurement (in terms of Δ) were found to be −28% (M1), +27% (M9), and +29% (M8). Underestimation of the model by 28% occurred during the first measurement period M1 in January 2015 in Iffwil for the measurement point at *D* = +40 m. This measurement point was close to a manhole cover made of cast-iron and concrete, as described in the methods section. As a consequence, the measurement point was moved by 5 m to *D* = +35 m for the later measurements.

The other large discrepancies between model and measurement occurred at the most distant measurement points at *D* = ±80 m in Iffwil (M9) and Wiler (M8).

To explore the relative deviation between model and measurement in relation to distance from the centre of the line, the data points were divided into two groups: “Near points” are the innermost three with orthogonal distances D = 0 and ±10 m; “far points” are the outer four at distances between 35 m and 80 m. For the Models A and B based on DHM25, the offset M(Δ) is considerably larger for near points than for far points at both measurement locations (Table 3). However, the opposite pattern is seen for the standard deviation between model and measurement. With the more precise terrain model used in Models C and D, the mean error M(Δ) for near-axis points becomes markedly smaller for both measurement locations (Table 4). For distant points, improvements compared to Models A and B are mainly seen in Wiler. The standard deviation is little affected by the choice of the terrain model, except for near points in Iffwil, where a reduction of the standard deviation is observed.

## 4. Discussion

The validation study has shown that the time-averaged magnetic field can be modelled with relative precision in the percent range.

Both the measurement, and, given the necessary input data, the computation of magnetic fields from overhead power lines are in principle straightforward, and the measurements and calculations generally agree well. An example with very detailed input (currents measured on individual phases and the shield wire) was reported by Swanson, 1995 [22]: the comparison, for a profile perpendicular to the line, gave agreement with a maximum error of ±7% ± 1 nT between measurement and calculation. While this comparison was for a 30 min period with constant currents, in our study, we also compare the temporal averages for longer periods with varying currents, and in addition have to evaluate the influence of simplifications, such as the constant value for the line sag and the use of symmetric currents. The explicit consideration of the effect of diurnal and seasonal variation in load flow is also a main difference from previous studies [20,23], where annual averages of currents have mostly been used for magnetic field calculations.

The precision of our results is of the same order as in [22]. For points near the line axis, it depends sensitively on the precision of the numerical terrain model. Given reliable height information and a reliable terrain model, the average relative precision (for a collection of points) is in the order of less than five per cent and standard deviation between measurements and modelling was less than ten per cent. The largest errors result from uncertainties in the coordinates, i.e., the x-y-coordinates, the height (both of conductors and terrain), and the line sag. The fact that the difference between the coarse and the fine terrain model is most pronounced for near points is to be expected, as the distance to the conductors is most strongly influenced by the height, and errors in the height act proportionally (or even stronger) on the calculated fields. For far points, the accuracy of the horizontal distance is more crucial, which is little dependent on the terrain model. The largest deviances between model and measurement were between −28% and +29%. One of these large deviations was caused by an artefact, which was due to an iron cover of a manhole close to the measurement device. This demonstrates that the magnetic fields are not only influenced by their primary sources (the high voltage line) alone, but may also be affected by conducting and/or magnetisable objects near the measurement points, where induced currents may occur in these objects and produce secondary magnetic fields. The reason for the other two large deviances remains unclear. A possible explanation may be due to the neglect of currents induced in the ground and shield wires and non-symmetric currents. Such currents are typically small (a few per cent of the symmetric currents), but as they produce fields that fall off less rapidly with distance than the fields from the symmetric currents, the fields from these unbalanced currents become relatively more important at large distances, where these large deviances were observed. Swanson [22] has included these unbalanced currents in the calculation. His Figure 11 shows that (for the line studied) the influence of the unbalanced currents remains negligible out to distances of approximately 100 m, where the magnetic field is reduced to about 98 nT or 4% of the value at line centre. Beyond that, their influence starts to grow, and at 200 m, they account for about one third of the calculated value of approx. 20 nT. A possibility to reduce the errors at large distances could therefore be to include the contributions from these induced currents. This could be done in principle, as the induced currents can be calculated from the symmetric currents and the electric and geometric properties of the conductors by applying the laws of induction. However, yet more information on the power line would have to be obtained and entered into the model, and it would only significantly affect the model in regions where the fields from the power line have already dropped to levels smaller than, e.g., the ambient levels found in typical households (ca. 50 nT, [21,24,25]), given that the observed discrepancies correspond to absolute errors of a mere 18 nT and 14 nT, respectively.

A strength of our study is the use of accurate data for line geometry and phase arrangement as well as load flow data with high temporal resolution. If such accurate data is not available, one is forced to make rough assumptions which would severely lower the model’s precision.

In order to verify that the conditions during our measurements were representative for the entire year, we have compared histograms of the meteorological data (temperature, wind, and global irradiation) from nearby stations of Meteo-Schweiz for our measurement periods to those for the entire year and concluded that they agreed well. Further, the temperature measurements made with the temperature loggers inside the boxes showed that the measurement devices always stayed in the specified range, even during cold winter nights when the outside temperature was below zero, due to the thermal insulation and buffering with PCM.

## 5. Conclusions

Repeated measurements with twelve measurement periods of 48 h distributed over a year at two measurement sites below two different power lines demonstrate the validity of our model to estimate time-averaged magnetic fields from power lines, taking into account both diurnal and seasonal variations of load flow and changing load flow directions. The accuracy of the model depends on the availability of accurate load and geometric data. In particular, the precision for near points depends strongly on the height information and can be improved by using an accurate digital terrain model. The application of the model on a national scale seems feasible; however, a considerable work effort would be required, as e.g., for Switzerland, the geometric data of some hundred overhead lines of ≥220 kV would have to be entered into the model.

## Figures and Tables

**Table 1:** Measurement periods at the two sites. “Begin” and “End” mark the times of the 48 h of data used for the evaluation.

Measurement Nr.	Site	Begin	End
M1	Iffwil	20 January 2015 13:30	22 January 2015 13:30
M2	Wiler	17 February 2015 14:00	19 February 2015 14:00
M3	Iffwil	24 March 2015 12:30	26 March 2015 12:30
M4	Wiler	8 April 2015 12:30	10 April 2015 12:30
M5	Iffwil	26 May 2015 14:00	28 May 2015 14:00
M6	Wiler	2 June 2015 14:00	04 June 2015 14:00
M7	Iffwil	7 July 2015 10:30	9 July 2015 10:30
M8	Wiler	28 July 2015 11:00	30 July 2015 11:00
M9	Iffwil	8 September 2015 12:30	10 September 2015 12:30
M10	Wiler	15 September 2015 13:00	17 September 2015 13:00
M11	Iffwil	27 October 2015 13:15	29 October 2015 13:15
M12	Wiler	8 December 2015 14:00	10 December 2015 14:00

**Table 2:** Mean M(Δ) and standard deviation σ(Δ) of the difference between model and measurements for the models with the DHM25 (Models A and B) compared to those with the DHM5 (Models C and D).

		Iffwil		Wiler	
		M(Δ)	σ(Δ)	M(Δ)	σ(Δ)
Model A (for $\bar{B}$)	DHM25	−0.04	0.11	0.07	0.08
Model B (for $B_{\mathrm{RMS}}$)		−0.04	0.11	0.08	0.08
Model C (for $\bar{B}$)	DHM5	0.02	0.09	−0.01	0.08
Model D (for $B_{\mathrm{RMS}}$)		0.02	0.09	−0.01	0.07

**Table 3:** Statistics (mean M(Δ) and standard deviation σ(Δ)) of the difference between model and measurement, separately for near and far points for models with the DHM25.

	Near Points, |D|≤10 m				Far Points, |D|≥35 m			
	Model A (for $\bar{B}$)		Model B (for $B_{\mathrm{RMS}}$)		Model A (for $\bar{B}$)		Model B (for $B_{\mathrm{RMS}}$)	
Site	M(Δ)	σ(Δ)	M(Δ)	σ(Δ)	M(Δ)	σ(Δ)	M(Δ)	σ(Δ)
Iffwil	−0.11	0.08	−0.12	0.07	0.03	0.13	0.03	0.12
Wiler	0.11	0.05	0.12	0.05	0.05	0.10	0.05	0.09

**Table 4:** Statistics (mean M(Δ) and standard deviation σ(Δ)) of the difference between model and measurement, separately for near and far points for models with the DHM5.

	Near Points, |D|≤10 m				Far Points, |D|≥35 m			
	Model C (for $\bar{B}$)		Model D (for $B_{\mathrm{RMS}}$)		Model C (for $\bar{B}$)		Model D (for $B_{\mathrm{RMS}}$)	
Site	M(Δ)	σ(Δ)	M(Δ)	σ(Δ)	M(Δ)	σ(Δ)	M(Δ)	σ(Δ)
Iffwil	0.01	0.04	0.01	0.04	0.03	0.12	0.04	0.12
Wiler	−0.03	0.05	−0.03	0.05	0.02	0.09	0.02	0.09

## Supplementary Materials

The following is available online at www.mdpi.com/1660-4601/14/9/949/s1, Table S1–S12: Comparison of model and measurements.

## Appendix A

**Table A1:** Technical data of the magnetic field measurement devices.

	*Emdex II*	*ESTEC DL-MW 10s*	*ESTEC EMLog 2e*
Manufacturer	EMDEX-LLC (www.emdex-llc.com)	ESTEC (www.estec.de)	
Measurement range	300 μT	130 μT	10 μT
Resolution	10 nT	10 nT	1 nT
Precision	±3% (overall) ±10% (worst case)	±3%, ±10 nT (one axis)	±3%, ±1 nT (one axis)
Axis-directions	*Y*-axis parallel to long side of devices	*X*-axis parallel to long side of devices	
Frequency bands	Broadband: 40–800 Hz Harmonics: 100–800 Hz	5–30 Hz 37–2000 Hz	
Data rate	1.5–327 s	1 s	
Battery-capacity ^1^	ca. 90 h	7 days	
Number of data points, Recording-period ^1^	15,000 <62 h	5,500,000 7 days	
Power source	9V Alkaline battery	Li-Ion, chargeable via USB-cable	
Data storage	Data deleted on power-off	Permanent storage	
Operating temperature range	0–60 °C	0–40 °C	

Data according to the data sheets of the manufacturers ^1^ in the operating mode used during the measurements.
