# Supplementary material for: Exposure Modelling of Extremely Low-Frequency Magnetic Fields from Overhead Power Lines and Its Validation by Measurements
Source: Int J Environ Res Public Health. 2017 Aug 23;14(9):949. doi: 10.3390/ijerph14090949 (PMC5615486; doi:10.3390/ijerph14090949)
Supplement: Supplementary file 1 [file ijerph-14-00949-s001.docx]

Supplementary Material

Exposure Modelling of Extremely Low-Frequency Magnetic Fields from Overhead Power Lines and its Validation by Measurements

Alfred Bürgi ^1,†^, Sanjay Sagar ^2,3,†^, Benjamin Struchen ^2,3^, Stefan Joss ^4^ and Martin Röösli ^2,3,^*

^1^ ARIAS umwelt.forschung.beratung gmbh, Gutenbergstrasse 40B, 3011 Bern, Switzerland; alfred.buergi@arias.ch

^2^ Swiss Tropical and Public Health Institute, Department of Epidemiology and Public Health, Socinstrasse 57, 4051 Basel, Switzerland; sanjay.sagar@unibas.ch (S.S.); benjamin.struchen@unibas.ch (B.S.)

^3^ University of Basel, Petersplatz 1, 4051 Basel, Switzerland

^4^ Federal Office for the Environment (FOEN), 3003 Bern, Switzerland; stefan.joss@bafu.admin.ch

***** Correspondence: martin.roosli@unibas.ch; Tel.: +41-61-284-83-83

† Joint first authors.

This supplementary material contains all tables with the comparison of model and measurements.

Content of the tables:

- Measurement number (M1, M2, etc.)
- Measurement device (Emdex 1, Emdex 2, …)
- $D$: Orthogonal distance from line axis (in m)
- $\bar{\boldsymbol{B}}$: Average absolute value of magnetic flux density ( in μT), measured and modelled
- $\boldsymbol{B}_{\boldsymbol{RMS}}$: Root-mean-square value of magnetic flux density ( in μT), measured and modeled
- $\Delta:= \frac{B \left( \mathrm{model} \right)-B(\mathrm{measured})}{B(\mathrm{measured})}$ : Relative error of model, $B$ is the appropriate average ($\bar{\boldsymbol{B}}$ or $\boldsymbol{B}_{\boldsymbol{RMS}}$), depending on the model

The four model variants are:

- Model A: Gives the arithmetic average $\bar{B}$ using the the 25-m-resolution terrain model DHM25
- Model B: Gives the RMS-mean $B_{\mathrm{RMS}}$ using the the 25-m-resolution terrain model DHM25
- Model C: Gives the arithmetic average $\bar{B}$ using the the 5-m-resolution terrain model DHM5
- Model D: Gives the RMS-mean $B_{\mathrm{RMS}}$ using the the 5-m-resolution terrain model DHM5

# Measurements in Iffwil

**Table S**1: Comparison of measurement and model for measurement M1 in Iffwil (Units: D in m, B in μT).

| Measure-ment M1 |  | Measurement | | Model A | | Model B | | Model C | | Model D | |
| --- | --- | --- | --- | --- | --- | --- | --- | --- | --- | --- | --- |
| Jan 2015 | $D$ | $\bar{\boldsymbol{B}}$ | $\boldsymbol{B}_{\boldsymbol{RMS}}$ | $\bar{\boldsymbol{B}}$ | $\boldsymbol{\Delta}$ | $\boldsymbol{B}_{\boldsymbol{RMS}}$ | $\boldsymbol{\Delta}$ | $\bar{\boldsymbol{B}}$ | $\boldsymbol{\Delta}$ | $\boldsymbol{B}_{\boldsymbol{RMS}}$ | $\boldsymbol{\Delta}$ |
| Emdex 1 | 0 | 1.220 | 1.338 | 1.030 | -16% | 1.140 | -15% | 1.187 | -3% | 1.325 | -1% |
| Emdex 2 | 10 | 0.795 | 0.849 | 0.664 | -16% | 0.719 | -15% | 0.735 | -7% | 0.801 | -6% |
| Emdex 3 | 20 | 0.406 | 0.427 | 0.349 | -14% | 0.372 | -13% | 0.366 | -10% | 0.391 | -8% |
| Emdex 4 | 40 | 0.166 | 0.175 | 0.120 | -28% | 0.129 | -26% | 0.121 | -27% | 0.130 | -26% |
| Estec 5 | 80 | 0.034 | 0.036 | 0.034 | 0% | 0.037 | 2% | 0.034 | -1% | 0.037 | 2% |
| Emdex 6 | -10 | 1.129 | 1.218 | 1.020 | -10% | 1.113 | -9% | 1.169 | 4% | 1.282 | 5% |
| Emdex 7 | -20 | 0.650 | 0.686 | 0.622 | -4% | 0.664 | -3% | 0.674 | 4% | 0.720 | 5% |
| Emdex 8 | -40 | 0.204 | 0.209 | 0.203 | 0% | 0.211 | 1% | 0.207 | 1% | 0.215 | 3% |
| Estec 9 | -80 | 0.049 | 0.049 | 0.049 | 0% | 0.051 | 3% | 0.049 | 0% | 0.051 | 3% |

**T**able S2: Comparison of measurement and model for measurement M3 in Iffwil (Units: D in m, B in μT).

| Measure-ment M3 |  | Measurement | | Model A | | Model B | | Model C | | Model D | |
| --- | --- | --- | --- | --- | --- | --- | --- | --- | --- | --- | --- |
| Mar 2015 | $D$ | $\bar{\boldsymbol{B}}$ | $\boldsymbol{B}_{\boldsymbol{RMS}}$ | $\bar{\boldsymbol{B}}$ | $\boldsymbol{\Delta}$ | $\boldsymbol{B}_{\boldsymbol{RMS}}$ | $\boldsymbol{\Delta}$ | $\bar{\boldsymbol{B}}$ | $\boldsymbol{\Delta}$ | $\boldsymbol{B}_{\boldsymbol{RMS}}$ | $\boldsymbol{\Delta}$ |
| Emdex 1 | 0 | 1.303 | 1.348 | 1.148 | -12% | 1.181 | -12% | 1.326 | 2% | 1.367 | 1% |
| Emdex 2 | 10 | 0.802 | 0.830 | 0.716 | -11% | 0.737 | -11% | 0.791 | -1% | 0.814 | -2% |
| Emdex 3 | 20 | 0.398 | 0.421 | 0.364 | -8% | 0.382 | -9% | 0.381 | -4% | 0.401 | -5% |
| Emdex 4 | 35 | 0.186 | 0.203 | 0.158 | -15% | 0.171 | -16% | 0.159 | -14% | 0.172 | -15% |
| Estec 5 | 80 | 0.034 | 0.036 | 0.037 | 10% | 0.041 | 11% | 0.037 | 10% | 0.041 | 11% |
| Emdex 6 | -10 | 1.294 | 1.342 | 1.126 | -13% | 1.160 | -14% | 1.294 | 0% | 1.334 | -1% |
| Emdex 7 | -20 | 0.732 | 0.760 | 0.667 | -9% | 0.688 | -9% | 0.718 | -2% | 0.741 | -3% |
| Emdex 8 | -40 | 0.221 | 0.230 | 0.221 | 0% | 0.229 | 0% | 0.225 | 2% | 0.233 | 1% |
| Estec 9 | -80 | 0.050 | 0.053 | 0.054 | 7% | 0.056 | 7% | 0.054 | 7% | 0.057 | 8% |

**T**able S3: Comparison of measurement and model for measurement M5 in Iffwil (Units: D in m, B in μT).

| Measure-ment M5 |  | Measurement | | Model A | | Model B | | Model C | | Model D | |
| --- | --- | --- | --- | --- | --- | --- | --- | --- | --- | --- | --- |
| May 2015 | $D$ | $\bar{\boldsymbol{B}}$ | $\boldsymbol{B}_{\boldsymbol{RMS}}$ | $\bar{\boldsymbol{B}}$ | $\boldsymbol{\Delta}$ | $\boldsymbol{B}_{\boldsymbol{RMS}}$ | $\boldsymbol{\Delta}$ | $\bar{\boldsymbol{B}}$ | $\boldsymbol{\Delta}$ | $\boldsymbol{B}_{\boldsymbol{RMS}}$ | $\boldsymbol{\Delta}$ |
| Emdex 1 | 0 | 1.254 | 1.364 | 1.122 | -11% | 1.206 | -12% | 1.283 | 2% | 1.389 | 2% |
| Emdex 2 | 10 | 1.018 | 1.100 | 0.912 | -10% | 0.982 | -11% | 1.024 | 1% | 1.107 | 1% |
| Emdex 3 | 20 | 0.564 | 0.609 | 0.523 | -7% | 0.564 | -7% | 0.556 | -1% | 0.600 | -1% |
| Emdex 4 | 35 | 0.261 | 0.280 | 0.234 | -10% | 0.252 | -10% | 0.236 | -10% | 0.255 | -9% |
| Estec 5 | 80 | 0.044 | 0.046 | 0.049 | 12% | 0.053 | 15% | 0.049 | 13% | 0.053 | 14% |
| Emdex 6 | -10 | 1.000 | 1.084 | 0.916 | -8% | 0.972 | -10% | 1.032 | 3% | 1.099 | 1% |
| Emdex 7 | -20 | 0.529 | 0.579 | 0.521 | -2% | 0.555 | -4% | 0.555 | 5% | 0.591 | 2% |
| Emdex 8 | -40 | 0.165 | 0.185 | 0.176 | 6% | 0.190 | 3% | 0.178 | 8% | 0.193 | 4% |
| Estec 9 | -80 | 0.041 | 0.046 | 0.047 | 14% | 0.051 | 11% | 0.047 | 14% | 0.051 | 11% |

**T**able S4: Comparison of measurement and model for measurement M7 in Iffwil (Units: D in m, B in μT).

| Measure-ment M7 |  | Measurement | | Model A | | Model B | | Model C | | Model D | |
| --- | --- | --- | --- | --- | --- | --- | --- | --- | --- | --- | --- |
| Jul 2015 | $D$ | $\bar{\boldsymbol{B}}$ | $\boldsymbol{B}_{\boldsymbol{RMS}}$ | $\bar{\boldsymbol{B}}$ | $\boldsymbol{\Delta}$ | $\boldsymbol{B}_{\boldsymbol{RMS}}$ | $\boldsymbol{\Delta}$ | $\bar{\boldsymbol{B}}$ | $\boldsymbol{\Delta}$ | $\boldsymbol{B}_{\boldsymbol{RMS}}$ | $\boldsymbol{\Delta}$ |
| Emdex 1 | 0 | 0.694 | 0.785 | 0.580 | -16% | 0.644 | -18% | 0.682 | -2% | 0.758 | -3% |
| Emdex 2 | 10 | 0.554 | 0.616 | 0.452 | -18% | 0.504 | -18% | 0.515 | -7% | 0.574 | -7% |
| Emdex 3 | 20 | 0.283 | 0.315 | 0.250 | -12% | 0.281 | -11% | 0.267 | -6% | 0.300 | -5% |
| Emdex 4 | 35 | 0.124 | 0.137 | 0.111 | -11% | 0.125 | -9% | 0.112 | -9% | 0.127 | -8% |
| Estec 5 | 80 | 0.022 | 0.024 | 0.023 | 3% | 0.026 | 10% | 0.023 | 4% | 0.026 | 10% |
| Emdex 6 | -10 | 0.475 | 0.538 | 0.431 | -9% | 0.482 | -10% | 0.491 | 3% | 0.551 | 2% |
| Emdex 7 | -20 | 0.231 | 0.263 | 0.227 | -2% | 0.258 | -2% | 0.243 | 5% | 0.276 | 5% |
| Emdex 8 | -40 | 0.071 | 0.083 | 0.075 | 6% | 0.088 | 6% | 0.077 | 9% | 0.090 | 8% |
| Estec 9 | -80 | 0.022 | 0.026 | 0.020 | -11% | 0.024 | -7% | 0.021 | -9% | 0.024 | -6% |

**T**able S5: Comparison of measurement and model for measurement M9 in Iffwil (Units: D in m, B in μT).

| Measure-ment M9 |  | Measurement | | Model A | | Model B | | Model C | | Model D | |
| --- | --- | --- | --- | --- | --- | --- | --- | --- | --- | --- | --- |
| Sep 2015 | $D$ | $\bar{\boldsymbol{B}}$ | $\boldsymbol{B}_{\boldsymbol{RMS}}$ | $\bar{\boldsymbol{B}}$ | $\boldsymbol{\Delta}$ | $\boldsymbol{B}_{\boldsymbol{RMS}}$ | $\boldsymbol{\Delta}$ | $\bar{\boldsymbol{B}}$ | $\boldsymbol{\Delta}$ | $\boldsymbol{B}_{\boldsymbol{RMS}}$ | $\boldsymbol{\Delta}$ |
| Emdex 1 | 0 | 1.663 | 1.711 | 1.518 | -9% | 1.552 | -9% | 1.708 | 3% | 1.749 | 2% |
| Emdex 2 | 10 | 1.255 | 1.311 | 1.179 | -6% | 1.216 | -7% | 1.300 | 4% | 1.345 | 3% |
| Emdex 3 | 20 | 0.740 | 0.774 | 0.726 | -2% | 0.747 | -3% | 0.766 | 4% | 0.789 | 2% |
| Emdex 4 | 35 | 0.380 | 0.394 | 0.358 | -6% | 0.365 | -7% | 0.362 | -5% | 0.369 | -6% |
| Estec 5 | 80 | 0.067 | 0.069 | 0.085 | 27% | 0.086 | 25% | 0.085 | 27% | 0.086 | 25% |
| Emdex 6 | -10 | 1.584 | 1.683 | 1.433 | -10% | 1.495 | -11% | 1.616 | 2% | 1.691 | 0% |
| Emdex 7 | -20 | 0.938 | 1.006 | 0.900 | -4% | 0.948 | -6% | 0.961 | 2% | 1.014 | 1% |
| Emdex 8 | -40 | 0.298 | 0.319 | 0.334 | 12% | 0.350 | 10% | 0.340 | 14% | 0.356 | 12% |
| Estec 9 | -80 | 0.077 | 0.082 | 0.092 | 19% | 0.095 | 16% | 0.092 | 19% | 0.095 | 17% |

**T**able S6: Comparison of measurement and model for measurement M11 in Iffwil (Units: D in m, B in μT).

| Measure-ment M11 |  | Measurement | | Model A | | Model B | | Model C | | Model D | |
| --- | --- | --- | --- | --- | --- | --- | --- | --- | --- | --- | --- |
| Oct 2015 | $D$ | $\bar{\boldsymbol{B}}$ | $\boldsymbol{B}_{\boldsymbol{RMS}}$ | $\bar{\boldsymbol{B}}$ | $\boldsymbol{\Delta}$ | $\boldsymbol{B}_{\boldsymbol{RMS}}$ | $\boldsymbol{\Delta}$ | $\bar{\boldsymbol{B}}$ | $\boldsymbol{\Delta}$ | $\boldsymbol{B}_{\boldsymbol{RMS}}$ | $\boldsymbol{\Delta}$ |
| Emdex 1 | 0 | 1.334 | 1.439 | 1.174 | -12% | 1.263 | -12% | 1.377 | 3% | 1.482 | 3% |
| Emdex 2 | 10 | 1.149 | 1.287 | 0.995 | -13% | 1.110 | -14% | 1.134 | -1% | 1.267 | -2% |
| Emdex 3 | 20 | 0.629 | 0.712 | 0.565 | -10% | 0.639 | -10% | 0.604 | -4% | 0.685 | -4% |
| Emdex 4 | 35 | 0.283 | 0.320 | 0.250 | -12% | 0.286 | -11% | 0.253 | -11% | 0.290 | -10% |
| Estec 5 | 80 | 0.045 | 0.051 | 0.051 | 12% | 0.059 | 15% | 0.051 | 12% | 0.058 | 15% |
| Emdex 6 | -10 | 0.783 | 0.813 | 0.763 | -3% | 0.792 | -3% | 0.857 | 9% | 0.887 | 9% |
| Emdex 7 | -20 | 0.358 | 0.377 | 0.373 | 4% | 0.387 | 3% | 0.392 | 9% | 0.407 | 8% |
| Emdex 8 | -40 | 0.116 | 0.123 | 0.133 | 15% | 0.141 | 14% | 0.135 | 16% | 0.142 | 16% |
| Estec 9 | -80 | 0.035 | 0.039 | 0.040 | 11% | 0.043 | 11% | 0.040 | 12% | 0.043 | 11% |

# Measurements in Wiler

**T**able S7: Comparison of measurement and model for measurement M2 in Wiler (Units: D in m, B in μT).

| Measure-ment M2 |  | Measurement | | Model A | | Model B | | Model C | | Model D | |
| --- | --- | --- | --- | --- | --- | --- | --- | --- | --- | --- | --- |
| Feb 2015 | $D$ | $\bar{\boldsymbol{B}}$ | $\boldsymbol{B}_{\boldsymbol{RMS}}$ | $\bar{\boldsymbol{B}}$ | $\boldsymbol{\Delta}$ | $\boldsymbol{B}_{\boldsymbol{RMS}}$ | $\boldsymbol{\Delta}$ | $\bar{\boldsymbol{B}}$ | $\boldsymbol{\Delta}$ | $\boldsymbol{B}_{\boldsymbol{RMS}}$ | $\boldsymbol{\Delta}$ |
| Emdex 1 | 0 | 0.687 | 0.719 | 0.812 | 18% | 0.851 | 18% | 0.705 | 3% | 0.741 | 3% |
| Emdex 2 | 10 | 0.688 | 0.747 | 0.776 | 13% | 0.851 | 14% | 0.691 | 0% | 0.758 | 1% |
| Emdex 3 | 20 | 0.538 | 0.600 | 0.569 | 6% | 0.650 | 8% | 0.526 | -2% | 0.601 | 0% |
| Emdex 4 | 40 | 0.266 | 0.302 | 0.251 | -6% | 0.300 | -1% | 0.244 | -8% | 0.291 | -4% |
| Estec 5 | 80 | 0.082 | 0.092 | 0.075 | -9% | 0.089 | -3% | 0.074 | -10% | 0.089 | -4% |
| Emdex 6 | -10 | 0.577 | 0.588 | 0.671 | 16% | 0.686 | 17% | 0.587 | 2% | 0.603 | 2% |
| Emdex 7 | -20 | 0.421 | 0.427 | 0.467 | 11% | 0.479 | 12% | 0.421 | 0% | 0.432 | 1% |
| Emdex 8 | -40 | --- | --- | 0.208 |  | 0.216 |  | 0.199 |  | 0.207 |  |
| Estec 9 | -80 | 0.064 | 0.065 | 0.066 | 3% | 0.070 | 7% | 0.065 | 2% | 0.069 | 6% |

**T**able S8: Comparison of measurement and model for measurement M4 in Wiler (Units: D in m, B in μT).

| Measure-ment M4 |  | Measurement | | Model A | | Model B | | Model C | | Model D | |
| --- | --- | --- | --- | --- | --- | --- | --- | --- | --- | --- | --- |
| Apr 2015 | $D$ | $\bar{\boldsymbol{B}}$ | $\boldsymbol{B}_{\boldsymbol{RMS}}$ | $\bar{\boldsymbol{B}}$ | $\boldsymbol{\Delta}$ | $\boldsymbol{B}_{\boldsymbol{RMS}}$ | $\boldsymbol{\Delta}$ | $\bar{\boldsymbol{B}}$ | $\boldsymbol{\Delta}$ | $\boldsymbol{B}_{\boldsymbol{RMS}}$ | $\boldsymbol{\Delta}$ |
| Emdex 1 | 0 | 0.955 | 0.984 | 1.084 | 14% | 1.112 | 13% | 0.970 | 2% | 0.994 | 1% |
| Emdex 2 | 10 | --- | --- | 1.113 |  | 1.155 |  | 1.005 |  | 1.040 |  |
| Emdex 3 | 20 | 0.845 | 0.883 | 0.892 | 6% | 0.925 | 5% | 0.830 | -2% | 0.859 | -3% |
| Emdex 4 | 40 | 0.434 | 0.456 | 0.439 | 1% | 0.452 | -1% | 0.427 | -2% | 0.439 | -4% |
| Estec 5 | 80 | 0.139 | 0.144 | 0.138 | -1% | 0.141 | -2% | 0.137 | -1% | 0.140 | -3% |
| Emdex 6 | -10 | 0.814 | 0.835 | 0.927 | 14% | 0.945 | 13% | 0.833 | 2% | 0.847 | 1% |
| Emdex 7 | -20 | 0.632 | 0.653 | 0.710 | 12% | 0.725 | 11% | 0.647 | 2% | 0.659 | 1% |
| Emdex 8 | -40 | 0.314 | 0.325 | 0.354 | 13% | 0.361 | 11% | 0.339 | 8% | 0.346 | 7% |
| Estec 9 | -80 | 0.104 | 0.108 | 0.119 | 14% | 0.121 | 12% | 0.118 | 13% | 0.120 | 11% |

**T**able S9: Comparison of measurement and model for measurement M6 in Wiler (Units: D in m, B in μT).

| Measure-ment M6 |  | Measurement | | Model A | | Model B | | Model C | | Model D | |
| --- | --- | --- | --- | --- | --- | --- | --- | --- | --- | --- | --- |
| Jun 2015 | $D$ | $\bar{\boldsymbol{B}}$ | $\boldsymbol{B}_{\boldsymbol{RMS}}$ | $\bar{\boldsymbol{B}}$ | $\boldsymbol{\Delta}$ | $\boldsymbol{B}_{\boldsymbol{RMS}}$ | $\boldsymbol{\Delta}$ | $\bar{\boldsymbol{B}}$ | $\boldsymbol{\Delta}$ | $\boldsymbol{B}_{\boldsymbol{RMS}}$ | $\boldsymbol{\Delta}$ |
| Emdex 1 | 0 | 1.328 | 1.432 | 1.450 | 9% | 1.562 | 9% | 1.228 | -8% | 1.320 | -8% |
| Emdex 2 | 10 | 1.085 | 1.225 | 1.121 | 3% | 1.267 | 3% | 0.993 | -8% | 1.116 | -9% |
| Emdex 3 | 20 | 0.701 | 0.821 | 0.689 | -2% | 0.809 | -1% | 0.639 | -9% | 0.746 | -9% |
| Emdex 4 | 40 | 0.280 | 0.335 | 0.268 | -4% | 0.315 | -6% | 0.260 | -7% | 0.306 | -9% |
| Estec 5 | 80 | 0.077 | 0.089 | 0.081 | 5% | 0.089 | 1% | 0.080 | 3% | 0.089 | 0% |
| Emdex 6 | -10 | 1.219 | 1.272 | 1.367 | 12% | 1.424 | 12% | 1.154 | -5% | 1.202 | -6% |
| Emdex 7 | -20 | 0.885 | 0.905 | 0.980 | 11% | 1.004 | 11% | 0.854 | -4% | 0.874 | -3% |
| Emdex 8 | -40 | 0.373 | 0.378 | 0.411 | 10% | 0.417 | 10% | 0.386 | 4% | 0.392 | 4% |
| Estec 9 | -80 | 0.094 | 0.097 | 0.112 | 19% | 0.115 | 18% | 0.111 | 17% | 0.113 | 16% |

**T**able S10: Comparison of measurement and model for measurement M8 in Wiler (Units: D in m, B in μT).

| Measure-ment M8 |  | Measurement | | Model A | | Model B | | Model C | | Model D | |
| --- | --- | --- | --- | --- | --- | --- | --- | --- | --- | --- | --- |
| Jul 2015 | $D$ | $\bar{\boldsymbol{B}}$ | $\boldsymbol{B}_{\boldsymbol{RMS}}$ | $\bar{\boldsymbol{B}}$ | $\boldsymbol{\Delta}$ | $\boldsymbol{B}_{\boldsymbol{RMS}}$ | $\boldsymbol{\Delta}$ | $\bar{\boldsymbol{B}}$ | $\boldsymbol{\Delta}$ | $\boldsymbol{B}_{\boldsymbol{RMS}}$ | $\boldsymbol{\Delta}$ |
| Emdex 1 | 0 | 0.870 | 0.967 | 0.962 | 11% | 1.076 | 11% | 0.812 | -7% | 0.906 | -6% |
| Emdex 2 | 10 | 0.774 | 0.883 | 0.786 | 2% | 0.914 | 3% | 0.693 | -10% | 0.801 | -9% |
| Emdex 3 | 20 | 0.527 | 0.619 | 0.502 | -5% | 0.601 | -3% | 0.463 | -12% | 0.552 | -11% |
| Emdex 4 | 40 | 0.219 | 0.260 | 0.198 | -9% | 0.236 | -9% | 0.192 | -12% | 0.228 | -12% |
| Estec 5 | 80 | 0.061 | 0.069 | 0.057 | -6% | 0.063 | -9% | 0.056 | -8% | 0.063 | -9% |
| Emdex 6 | -10 | 0.736 | 0.795 | 0.856 | 16% | 0.932 | 17% | 0.723 | -2% | 0.786 | -1% |
| Emdex 7 | -20 | 0.515 | 0.548 | 0.585 | 14% | 0.628 | 15% | 0.510 | -1% | 0.548 | 0% |
| Emdex 8 | -40 | 0.194 | 0.206 | 0.231 | 19% | 0.247 | 20% | 0.217 | 12% | 0.233 | 13% |
| Estec 9 | -80 | 0.047 | 0.052 | 0.061 | 29% | 0.066 | 27% | 0.060 | 28% | 0.065 | 25% |

**T**able S11: Comparison of measurement and model for measurement M10 in Wiler (Units: D in m, B in μT).

| Measure-ment M10 |  | Measurement | | Model A | | Model B | | Model C | | Model D | |
| --- | --- | --- | --- | --- | --- | --- | --- | --- | --- | --- | --- |
| Sep 2015 | $D$ | $\bar{\boldsymbol{B}}$ | $\boldsymbol{B}_{\boldsymbol{RMS}}$ | $\bar{\boldsymbol{B}}$ | $\boldsymbol{\Delta}$ | $\boldsymbol{B}_{\boldsymbol{RMS}}$ | $\boldsymbol{\Delta}$ | $\bar{\boldsymbol{B}}$ | $\boldsymbol{\Delta}$ | $\boldsymbol{B}_{\boldsymbol{RMS}}$ | $\boldsymbol{\Delta}$ |
| Emdex 1 | 0 | 0.767 | 0.783 | 0.839 | 9% | 0.862 | 10% | 0.707 | -8% | 0.727 | -7% |
| Emdex 2 | 10 | 0.708 | 0.719 | 0.735 | 4% | 0.751 | 4% | 0.644 | -9% | 0.659 | -8% |
| Emdex 3 | 20 | 0.492 | 0.501 | 0.493 | 0% | 0.504 | 1% | 0.452 | -8% | 0.462 | -8% |
| Emdex 4 | 40 | 0.195 | 0.200 | 0.194 | 0% | 0.199 | 0% | 0.187 | -4% | 0.193 | -4% |
| Estec 5 | 80 | 0.049 | 0.051 | 0.050 | 3% | 0.052 | 2% | 0.050 | 2% | 0.052 | 1% |
| Emdex 6 | -10 | 0.638 | 0.658 | 0.702 | 10% | 0.732 | 11% | 0.594 | -7% | 0.619 | -6% |
| Emdex 7 | -20 | 0.433 | 0.451 | 0.459 | 6% | 0.486 | 8% | 0.402 | -7% | 0.424 | -6% |
| Emdex 8 | -40 | 0.167 | 0.175 | 0.175 | 5% | 0.186 | 6% | 0.165 | -1% | 0.175 | 0% |
| Estec 9 | -80 | 0.041 | 0.043 | 0.046 | 11% | 0.048 | 11% | 0.045 | 9% | 0.048 | 10% |

**T**able S12: Comparison of measurement and model for measurement M12 in Wiler (Units: D in m, B in μT).

| Measure-ment M12 |  | Measurement | | Model A | | Model B | | Model C | | Model D | |
| --- | --- | --- | --- | --- | --- | --- | --- | --- | --- | --- | --- |
| Dec 2015 | $D$ | $\bar{\boldsymbol{B}}$ | $\boldsymbol{B}_{\boldsymbol{RMS}}$ | $\bar{\boldsymbol{B}}$ | $\boldsymbol{\Delta}$ | $\boldsymbol{B}_{\boldsymbol{RMS}}$ | $\boldsymbol{\Delta}$ | $\bar{\boldsymbol{B}}$ | $\boldsymbol{\Delta}$ | $\boldsymbol{B}_{\boldsymbol{RMS}}$ | $\boldsymbol{\Delta}$ |
| Emdex 1 | 0 | 0.977 | 1.082 | 1.158 | 18% | 1.273 | 18% | 1.006 | 3% | 1.105 | 2% |
| Emdex 2 | 10 | 1.021 | 1.162 | 1.158 | 13% | 1.306 | 12% | 1.029 | 1% | 1.158 | 0% |
| Emdex 3 | 20 | 0.822 | 0.949 | 0.874 | 6% | 0.999 | 5% | 0.806 | -2% | 0.920 | -3% |
| Emdex 4 | 40 | 0.385 | 0.450 | 0.392 | 2% | 0.454 | 1% | 0.380 | -1% | 0.440 | -2% |
| Estec 5 | 80 | 0.114 | 0.133 | 0.113 | -1% | 0.131 | -1% | 0.112 | -2% | 0.130 | -2% |
| Emdex 6 | -10 | --- | --- | 0.906 |  | 0.966 |  | 0.796 |  | 0.851 |  |
| Emdex 7 | -20 | 0.536 | 0.568 | 0.602 | 12% | 0.634 | 12% | 0.546 | 2% | 0.575 | 1% |
| Emdex 8 | -40 | 0.235 | 0.247 | 0.257 | 10% | 0.270 | 9% | 0.247 | 5% | 0.260 | 5% |
| Estec 9 | -80 | 0.075 | 0.080 | 0.082 | 9% | 0.087 | 9% | 0.081 | 8% | 0.086 | 8% |
