# Supplementary material for: Exposure Modelling of Extremely Low-Frequency Magnetic Fields from Overhead Power Lines and Its Validation by Measurements
Source: Int J Environ Res Public Health. 2017 Aug 23;14(9):949. doi: 10.3390/ijerph14090949 (PMC5615486; doi:10.3390/ijerph14090949)
Supplement: Supplementary file 2 [file ijerph-14-00949-s002.pdf]

International Journal of
Environmental Research and Public Health

MDPI

Supplementary Material

Exposure Modelling of Extremely Low-Frequency Magnetic Fields from Overhead Power Lines and its Validation by Measurements

Alfred Bürgi [1,†], Sanjay Sagar [2,3,†], Benjamin Struchen [2,3], Stefan Joss [4] and Martin Röösli [2,3,*]

1 ARIAS umwelt.forschung.beratung gmbh, Gutenbergstrasse 40B, 3011 Bern, Switzerland; alfred.buergi@arias.ch
2 Swiss Tropical and Public Health Institute, Department of Epidemiology and Public Health, Socinstrasse 57, 4051 Basel, Switzerland; sanjay.sagar@unibas.ch (S.S.); benjamin.struchen@unibas.ch (B.S.)
3 University of Basel, Petersplatz 1, 4051 Basel, Switzerland
4 Federal Office for the Environment (FOEN), 3003 Bern, Switzerland; stefan.joss@bafu.admin.ch
* Correspondence: martin.roosli@unibas.ch; Tel.: +41-61-284-83-83
† Joint first authors.

This supplementary material contains all tables with the comparison of model and measurements

Content of the tables:

- Measurement number (M1, M2, etc.)
- Measurement device (Emdex 1, Emdex 2, …)
- D: Orthogonal distance from line axis (in m)
- $\overline{\boldsymbol{B}}$: Average absolute value of magnetic flux density (in µT), measured and modelled
- $\boldsymbol{B_{RMS}}$: Root-mean-square value of magnetic flux density (in µT), measured and modeled
- $\Delta := \frac{B\text{ (model)}-B\text{(measured)}}{B\text{(measured)}}$: Relative error of model, B is the appropriate average ($\overline{\boldsymbol{B}}$ or $\boldsymbol{B_{RMS}}$), depending on the model

The four model variants are:

- Model A: Gives the arithmetic average $\overline{\mathrm{B}}$ using the the 25-m-resolution terrain model DHM25
- Model B: Gives the RMS-mean $\mathrm{B_{RMS}}$ using the the 25-m-resolution terrain model DHM25
- Model C: Gives the arithmetic average $\overline{\mathrm{B}}$ using the the 5-m-resolution terrain model DHM5
- Model D: Gives the RMS-mean $\mathrm{B_{RMS}}$ using the the 5-m-resolution terrain model DHM5

Measurements in Iffwil

Table S1: Comparison of measurement and model for measurement M1 in Iffwil (Units: D in m, B in μT).

Measure-ment M1		***Measurement***		***Model A***		***Model B***		***Model C***		***Model D***	
Jan 2015	*D*	$\overline{B}$	B_{RMS}	$\overline{B}$	Δ	B_{RMS}	Δ	$\overline{B}$	Δ	B_{RMS}	Δ
Emdex 1	0	1.220	1.338	1.030	-16%	1.140	-15%	1.187	-3%	1.325	-1%
Emdex 2	10	0.795	0.849	0.664	-16%	0.719	-15%	0.735	-7%	0.801	-6%
Emdex 3	20	0.406	0.427	0.349	-14%	0.372	-13%	0.366	-10%	0.391	-8%
Emdex 4	40	0.166	0.175	0.120	-28%	0.129	-26%	0.121	-27%	0.130	-26%
Estec 5	80	0.034	0.036	0.034	0%	0.037	2%	0.034	-1%	0.037	2%
Emdex 6	-10	1.129	1.218	1.020	-10%	1.113	-9%	1.169	4%	1.282	5%
Emdex 7	-20	0.650	0.686	0.622	-4%	0.664	-3%	0.674	4%	0.720	5%
Emdex 8	-40	0.204	0.209	0.203	0%	0.211	1%	0.207	1%	0.215	3%
Estec 9	-80	0.049	0.049	0.049	0%	0.051	3%	0.049	0%	0.051	3%

Table S2: Comparison of measurement and model for measurement M3 in Iffwil (Units: D in m, B in μT).

Measure-ment M3		***Measurement***		***Model A***		***Model B***		***Model C***		***Model D***	
Mar 2015	*D*	$\overline{B}$	B_{RMS}	$\overline{B}$	Δ	B_{RMS}	Δ	$\overline{B}$	Δ	B_{RMS}	Δ
Emdex 1	0	1.303	1.348	1.148	-12%	1.181	-12%	1.326	2%	1.367	1%
Emdex 2	10	0.802	0.830	0.716	-11%	0.737	-11%	0.791	-1%	0.814	-2%
Emdex 3	20	0.398	0.421	0.364	-8%	0.382	-9%	0.381	-4%	0.401	-5%
Emdex 4	35	0.186	0.203	0.158	-15%	0.171	-16%	0.159	-14%	0.172	-15%
Estec 5	80	0.034	0.036	0.037	10%	0.041	11%	0.037	10%	0.041	11%
Emdex 6	-10	1.294	1.342	1.126	-13%	1.160	-14%	1.294	0%	1.334	-1%
Emdex 7	-20	0.732	0.760	0.667	-9%	0.688	-9%	0.718	-2%	0.741	-3%
Emdex 8	-40	0.221	0.230	0.221	0%	0.229	0%	0.225	2%	0.233	1%
Estec 9	-80	0.050	0.053	0.054	7%	0.056	7%	0.054	7%	0.057	8%

Table S3: Comparison of measurement and model for measurement M5 in Iffwil (Units: D in m, B in μT).

Measure-ment M5		***Measurement***		***Model A***		***Model B***		***Model C***		***Model D***	
May 2015	*D*	$\bar{B}$	B_{RMS}	$\bar{B}$	Δ	B_{RMS}	Δ	$\bar{B}$	Δ	B_{RMS}	Δ
Emdex 1	0	1.254	1.364	1.122	-11%	1.206	-12%	1.283	2%	1.389	2%
Emdex 2	10	1.018	1.100	0.912	-10%	0.982	-11%	1.024	1%	1.107	1%
Emdex 3	20	0.564	0.609	0.523	-7%	0.564	-7%	0.556	-1%	0.600	-1%
Emdex 4	35	0.261	0.280	0.234	-10%	0.252	-10%	0.236	-10%	0.255	-9%
Estec 5	80	0.044	0.046	0.049	12%	0.053	15%	0.049	13%	0.053	14%
Emdex 6	-10	1.000	1.084	0.916	-8%	0.972	-10%	1.032	3%	1.099	1%
Emdex 7	-20	0.529	0.579	0.521	-2%	0.555	-4%	0.555	5%	0.591	2%
Emdex 8	-40	0.165	0.185	0.176	6%	0.190	3%	0.178	8%	0.193	4%
Estec 9	-80	0.041	0.046	0.047	14%	0.051	11%	0.047	14%	0.051	11%

Table S4: Comparison of measurement and model for measurement M7 in Iffwil (Units: D in m, B in μT).

Measure-ment M7		***Measurement***		***Model A***		***Model B***		***Model C***		***Model D***	
Jul 2015	*D*	$\bar{B}$	B_{RMS}	$\bar{B}$	Δ	B_{RMS}	Δ	$\bar{B}$	Δ	B_{RMS}	Δ
Emdex 1	0	0.694	0.785	0.580	-16%	0.644	-18%	0.682	-2%	0.758	-3%
Emdex 2	10	0.554	0.616	0.452	-18%	0.504	-18%	0.515	-7%	0.574	-7%
Emdex 3	20	0.283	0.315	0.250	-12%	0.281	-11%	0.267	-6%	0.300	-5%
Emdex 4	35	0.124	0.137	0.111	-11%	0.125	-9%	0.112	-9%	0.127	-8%
Estec 5	80	0.022	0.024	0.023	3%	0.026	10%	0.023	4%	0.026	10%
Emdex 6	-10	0.475	0.538	0.431	-9%	0.482	-10%	0.491	3%	0.551	2%
Emdex 7	-20	0.231	0.263	0.227	-2%	0.258	-2%	0.243	5%	0.276	5%
Emdex 8	-40	0.071	0.083	0.075	6%	0.088	6%	0.077	9%	0.090	8%
Estec 9	-80	0.022	0.026	0.020	-11%	0.024	-7%	0.021	-9%	0.024	-6%

Table S5: Comparison of measurement and model for measurement M9 in Iffwil (Units: D in m, B in µT).

Measure-ment M9		***Measurement***		***Model A***		***Model B***		***Model C***		***Model D***	
Sep 2015	D	$\bar{B}$	B_{RMS}	$\bar{B}$	Δ	B_{RMS}	Δ	$\bar{B}$	Δ	B_{RMS}	Δ
Emdex 1	0	1.663	1.711	1.518	-9%	1.552	-9%	1.708	3%	1.749	2%
Emdex 2	10	1.255	1.311	1.179	-6%	1.216	-7%	1.300	4%	1.345	3%
Emdex 3	20	0.740	0.774	0.726	-2%	0.747	-3%	0.766	4%	0.789	2%
Emdex 4	35	0.380	0.394	0.358	-6%	0.365	-7%	0.362	-5%	0.369	-6%
Estec 5	80	0.067	0.069	0.085	27%	0.086	25%	0.085	27%	0.086	25%
Emdex 6	-10	1.584	1.683	1.433	-10%	1.495	-11%	1.616	2%	1.691	0%
Emdex 7	-20	0.938	1.006	0.900	-4%	0.948	-6%	0.961	2%	1.014	1%
Emdex 8	-40	0.298	0.319	0.334	12%	0.350	10%	0.340	14%	0.356	12%
Estec 9	-80	0.077	0.082	0.092	19%	0.095	16%	0.092	19%	0.095	17%

Table S6: Comparison of measurement and model for measurement M11 in Iffwil (Units: D in m, B in µT).

Measure-ment M11		***Measurement***		***Model A***		***Model B***		***Model C***		***Model D***	
Oct 2015	D	$\bar{B}$	B_{RMS}	$\bar{B}$	Δ	B_{RMS}	Δ	$\bar{B}$	Δ	B_{RMS}	Δ
Emdex 1	0	1.334	1.439	1.174	-12%	1.263	-12%	1.377	3%	1.482	3%
Emdex 2	10	1.149	1.287	0.995	-13%	1.110	-14%	1.134	-1%	1.267	-2%
Emdex 3	20	0.629	0.712	0.565	-10%	0.639	-10%	0.604	-4%	0.685	-4%
Emdex 4	35	0.283	0.320	0.250	-12%	0.286	-11%	0.253	-11%	0.290	-10%
Estec 5	80	0.045	0.051	0.051	12%	0.059	15%	0.051	12%	0.058	15%
Emdex 6	-10	0.783	0.813	0.763	-3%	0.792	-3%	0.857	9%	0.887	9%
Emdex 7	-20	0.358	0.377	0.373	4%	0.387	3%	0.392	9%	0.407	8%
Emdex 8	-40	0.116	0.123	0.133	15%	0.141	14%	0.135	16%	0.142	16%
Estec 9	-80	0.035	0.039	0.040	11%	0.043	11%	0.040	12%	0.043	11%

Measurements in Wiler

Table S7: Comparison of measurement and model for measurement M2 in Wiler (Units: D in m, B in μT).

Measure-ment M2		***Measurement***		***Model A***		***Model B***		***Model C***		***Model D***	
Feb 2015	D	$\overline{B}$	B_{RMS}	$\overline{B}$	Δ	B_{RMS}	Δ	$\overline{B}$	Δ	B_{RMS}	Δ
Emdex 1	0	0.687	0.719	0.812	18%	0.851	18%	0.705	3%	0.741	3%
Emdex 2	10	0.688	0.747	0.776	13%	0.851	14%	0.691	0%	0.758	1%
Emdex 3	20	0.538	0.600	0.569	6%	0.650	8%	0.526	-2%	0.601	0%
Emdex 4	40	0.266	0.302	0.251	-6%	0.300	-1%	0.244	-8%	0.291	-4%
Estec 5	80	0.082	0.092	0.075	-9%	0.089	-3%	0.074	-10%	0.089	-4%
Emdex 6	-10	0.577	0.588	0.671	16%	0.686	17%	0.587	2%	0.603	2%
Emdex 7	-20	0.421	0.427	0.467	11%	0.479	12%	0.421	0%	0.432	1%
Emdex 8	-40	---	---	0.208		0.216		0.199		0.207	
Estec 9	-80	0.064	0.065	0.066	3%	0.070	7%	0.065	2%	0.069	6%

Table S8: Comparison of measurement and model for measurement M4 in Wiler (Units: D in m, B in μT).

Measure-ment M4		***Measurement***		***Model A***		***Model B***		***Model C***		***Model D***	
Apr 2015	D	$\overline{B}$	B_{RMS}	$\overline{B}$	Δ	B_{RMS}	Δ	$\overline{B}$	Δ	B_{RMS}	Δ
Emdex 1	0	0.955	0.984	1.084	14%	1.112	13%	0.970	2%	0.994	1%
Emdex 2	10	---	---	1.113		1.155		1.005		1.040	
Emdex 3	20	0.845	0.883	0.892	6%	0.925	5%	0.830	-2%	0.859	-3%
Emdex 4	40	0.434	0.456	0.439	1%	0.452	-1%	0.427	-2%	0.439	-4%
Estec 5	80	0.139	0.144	0.138	-1%	0.141	-2%	0.137	-1%	0.140	-3%
Emdex 6	-10	0.814	0.835	0.927	14%	0.945	13%	0.833	2%	0.847	1%
Emdex 7	-20	0.632	0.653	0.710	12%	0.725	11%	0.647	2%	0.659	1%
Emdex 8	-40	0.314	0.325	0.354	13%	0.361	11%	0.339	8%	0.346	7%
Estec 9	-80	0.104	0.108	0.119	14%	0.121	12%	0.118	13%	0.120	11%

Table S9: Comparison of measurement and model for measurement M6 in Wiler (Units: D in m, B in μT).

Measure-ment M6		***Measurement***		***Model A***		***Model B***		***Model C***		***Model D***	
Jun 2015	D	$\overline{B}$	B_{RMS}	$\overline{B}$	Δ	B_{RMS}	Δ	$\overline{B}$	Δ	B_{RMS}	Δ
Emdex 1	0	1.328	1.432	1.450	9%	1.562	9%	1.228	-8%	1.320	-8%
Emdex 2	10	1.085	1.225	1.121	3%	1.267	3%	0.993	-8%	1.116	-9%
Emdex 3	20	0.701	0.821	0.689	-2%	0.809	-1%	0.639	-9%	0.746	-9%
Emdex 4	40	0.280	0.335	0.268	-4%	0.315	-6%	0.260	-7%	0.306	-9%
Estec 5	80	0.077	0.089	0.081	5%	0.089	1%	0.080	3%	0.089	0%
Emdex 6	-10	1.219	1.272	1.367	12%	1.424	12%	1.154	-5%	1.202	-6%
Emdex 7	-20	0.885	0.905	0.980	11%	1.004	11%	0.854	-4%	0.874	-3%
Emdex 8	-40	0.373	0.378	0.411	10%	0.417	10%	0.386	4%	0.392	4%
Estec 9	-80	0.094	0.097	0.112	19%	0.115	18%	0.111	17%	0.113	16%

Table S10: Comparison of measurement and model for measurement M8 in Wiler (Units: D in m, B in μT).

Measure-ment M8		***Measurement***		***Model A***		***Model B***		***Model C***		***Model D***	
Jul 2015	D	$\overline{B}$	B_{RMS}	$\overline{B}$	Δ	B_{RMS}	Δ	$\overline{B}$	Δ	B_{RMS}	Δ
Emdex 1	0	0.870	0.967	0.962	11%	1.076	11%	0.812	-7%	0.906	-6%
Emdex 2	10	0.774	0.883	0.786	2%	0.914	3%	0.693	-10%	0.801	-9%
Emdex 3	20	0.527	0.619	0.502	-5%	0.601	-3%	0.463	-12%	0.552	-11%
Emdex 4	40	0.219	0.260	0.198	-9%	0.236	-9%	0.192	-12%	0.228	-12%
Estec 5	80	0.061	0.069	0.057	-6%	0.063	-9%	0.056	-8%	0.063	-9%
Emdex 6	-10	0.736	0.795	0.856	16%	0.932	17%	0.723	-2%	0.786	-1%
Emdex 7	-20	0.515	0.548	0.585	14%	0.628	15%	0.510	-1%	0.548	0%
Emdex 8	-40	0.194	0.206	0.231	19%	0.247	20%	0.217	12%	0.233	13%
Estec 9	-80	0.047	0.052	0.061	29%	0.066	27%	0.060	28%	0.065	25%

Table S11: Comparison of measurement and model for measurement M10 in Wiler (Units: D in m, B in µT).

Measure-ment M10		***Measurement***		***Model A***		***Model B***		***Model C***		***Model D***	
Sep 2015	*D*	$\bar{B}$	B_{RMS}	$\bar{B}$	Δ	B_{RMS}	Δ	$\bar{B}$	Δ	B_{RMS}	Δ
Emdex 1	0	0.767	0.783	0.839	9%	0.862	10%	0.707	-8%	0.727	-7%
Emdex 2	10	0.708	0.719	0.735	4%	0.751	4%	0.644	-9%	0.659	-8%
Emdex 3	20	0.492	0.501	0.493	0%	0.504	1%	0.452	-8%	0.462	-8%
Emdex 4	40	0.195	0.200	0.194	0%	0.199	0%	0.187	-4%	0.193	-4%
Estec 5	80	0.049	0.051	0.050	3%	0.052	2%	0.050	2%	0.052	1%
Emdex 6	-10	0.638	0.658	0.702	10%	0.732	11%	0.594	-7%	0.619	-6%
Emdex 7	-20	0.433	0.451	0.459	6%	0.486	8%	0.402	-7%	0.424	-6%
Emdex 8	-40	0.167	0.175	0.175	5%	0.186	6%	0.165	-1%	0.175	0%
Estec 9	-80	0.041	0.043	0.046	11%	0.048	11%	0.045	9%	0.048	10%

Table S12: Comparison of measurement and model for measurement M12 in Wiler (Units: D in m, B in µT).

Measure-ment M12		***Measurement***		***Model A***		***Model B***		***Model C***		***Model D***	
Dec 2015	*D*	$\bar{B}$	B_{RMS}	$\bar{B}$	Δ	B_{RMS}	Δ	$\bar{B}$	Δ	B_{RMS}	Δ
Emdex 1	0	0.977	1.082	1.158	18%	1.273	18%	1.006	3%	1.105	2%
Emdex 2	10	1.021	1.162	1.158	13%	1.306	12%	1.029	1%	1.158	0%
Emdex 3	20	0.822	0.949	0.874	6%	0.999	5%	0.806	-2%	0.920	-3%
Emdex 4	40	0.385	0.450	0.392	2%	0.454	1%	0.380	-1%	0.440	-2%
Estec 5	80	0.114	0.133	0.113	-1%	0.131	-1%	0.112	-2%	0.130	-2%
Emdex 6	-10	---	---	0.906		0.966		0.796		0.851	
Emdex 7	-20	0.536	0.568	0.602	12%	0.634	12%	0.546	2%	0.575	1%
Emdex 8	-40	0.235	0.247	0.257	10%	0.270	9%	0.247	5%	0.260	5%
Estec 9	-80	0.075	0.080	0.082	9%	0.087	9%	0.081	8%	0.086	8%
